# FedSMOTE-DP: Privacy-Aware Federated Ensemble Learning for Intrusion Detection in IoMT Networks

**DOI:** 10.3390/s26051592

**Published:** 2026-03-03

**Authors:** Theyab Alsolami, Mohammad Ilyas

**Affiliations:** 1 Department of Electrical Engineering and Computer Science, Florida Atlantic University, 777 Glades Road, Boca Raton, FL 33431, USA; 2 College of Computer, Najran University, Najran 55461, Saudi Arabia

**Keywords:** Internet of Medical Things (IoMT), intrusion detection system (IDS), Federated Learning (FL), ensemble learning, differential privacy, secure aggregation, cybersecurity, machine learning, privacy preservation

## Abstract

The Internet of Medical Things (IoMT) transforms healthcare through interconnected medical devices but faces significant cybersecurity threats, particularly intrusion and exfiltration attacks. Centralized intrusion detection systems (IDSs) require data aggregation, presenting privacy and scalability risks. This paper proposes FedEnsemble-DP, a privacy-aware Federated Learning (FL) framework for decentralized intrusion detection in IoMT networks. The framework integrates three data balancing scenarios (Raw Imbalanced, Local SMOTE, Centralized SMOTE) with Differential Privacy (DP) and Secure Aggregation mechanisms. Extensive experiments on WUSTL-EHMS-2020 and CIC-IoMT-2024 datasets under non-IID settings (Dirichlet α = 0.3) demonstrate that models with strong privacy guarantees (ε = 3.0) frequently match or exceed non-private baselines. Key findings show Local SMOTE with ε = 3.0 achieved 94.60% accuracy and 0.9598 AUC, while Raw Imbalanced with ε = 3.0 attained 94.50% accuracy and 0.9494 AUC. Even with strict privacy (ε = 3.0), these results surpassed the non-private baseline (93.20% accuracy) in the raw scenario. Centralized SMOTE showed effectiveness but introduced training instability. These results indicate that local data balancing combined with calibrated DP noise can yield high detection performance while preserving privacy, effectively bridging security-performance and data confidentiality requirements in distributed healthcare networks.

## 1. Introduction

**Figure 1:**
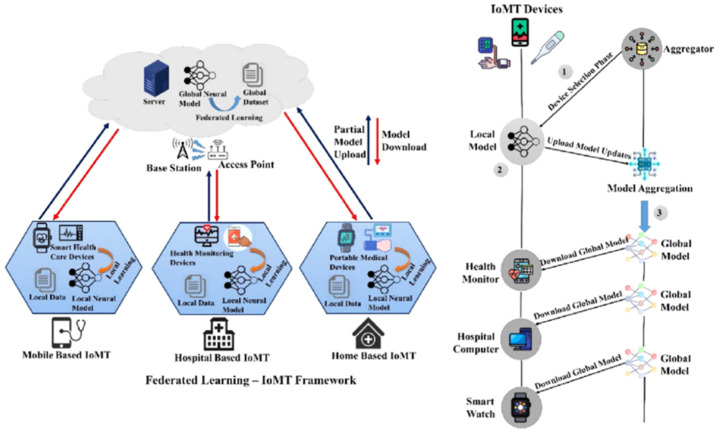
FL and SMOTE analysis and IoMT architecture.

The IoMT has essentially altered the current healthcare model by supporting uninterrupted patient attention, automatic diagnostics, and distance therapeutic interventions. This networked ecosystem, nevertheless, broadens the attacker target of malicious professionals, and healthcare networks are the ideal targets of advanced cyber-attacks that harm the safety and privacy of patients [1,2,3]. The medical data is sensitive and has strict regulations such as HIPAA and GDPR, which require an intrusion detection system (IDS) that is not only correct in its results but also privacy-saving. The centralized IDS designs using traditional models necessitating the aggregation of raw network data are inconsistent with these privacy requirements and pose great risks of breaches of data and single points of failure [4,5,6]. Figure 1 shows the structure of the IoMT environment with illustrated features [6,7].

By allowing joint model training among distributed devices without the exchange of raw data, Federated Learning (FL) truly offers a paradigm shift by meeting the requirements of the decentralized nature of IoMT and fundamental privacy principles. Nonetheless, using FL to perform intrusion detection in realistic IoMT systems creates acute problems, such as extreme imbalance in the number of attacks and the reduced performance inherent to implementing robust privacy protection mechanisms, such as Differential Privacy (DP). A trade-off between model utility and preservation of privacy has posed a major challenge, and in most cases, a compromise must be made to the entire system, affecting the security efficacy of the system [8,9]. Table 1 below defines the acronyms used throughout the manuscript.

To address these particular challenges to successful intrusion detection in IoMT networks, this paper presents and critically analyzes an optimized FL-based method that overcomes these particular challenges. The proposed methodology is a systematic study of the interaction of data balance strategies and privacy budgets. It uses the Synthetic Minority Over-sampling Technique (SMOTE) used at the various levels of federation; centrally and per-client, to address the imbalance in classes. It also, concurrently, experiments on privacy budgets (ϵ), of 3.0 and 10.0, and a non-private control (ϵ = inf), to measure the privacy-performance trade-off [10,11].

Experimental research of a realistic dataset of an IoMT network shows that the suggested method can be effective at preserving a high detection rate despite severe privacy limitations. As an example, in the Raw Imbalanced scenario where the privacy budget (ϵ = 3.0) is strict, the model obtained an end test accuracy of 94.50% and an F1-Score of 0.9450, which indicates good learning in spite of unstable validation performance throughout training [12,13,14]. It is important to note that with the same level of privacy, per-client SMOTE produced an outstanding Area Under the Curve (AUC) of 0.9598 and a F1-Score of 0.9460, which demonstrated the applicability of the method in improving minority classes recall without compromising the privacy. Most importantly, the findings demonstrate that models that are tuned with DP (ϵ = 3.0, 10.0) can be as effective or even better than the non-private baseline that achieved a Raw Imbalanced accuracy of 93.20%. This shows that considerable privacy can be obtained without much utility being lost. Centralized SMOTE was effective, but more training instability was provoked, thus the significance of the selected balance approach in the FL loop [15,16,17].

As such, the work has a central role to play by illustrating a plausible and streamlined process of implementing privacy-aware FL in the IoMT security. It presents empirical results that indicate that with the co-construction of imbalance recovery and controlled noise injection, it is possible to create an IDS that does not violate strong data privacy policies and ensures that the high detection rate needed to protect important healthcare infrastructure is achieved. The sections below expound on the federated architecture, the experiment setups, and elaborate review of how various tradeoffs between the privacy and preprocessing strategies affect the overall security posture of the system [18,19].

Existing FL-based IDS frameworks often rely heavily on deep learning (DL) architectures such as convolutional neural networks (CNNs) and long short-term memory (LSTM) models [20,21,22]. Although these approaches achieve high accuracy, they are computationally expensive, resource-intensive, and less interpretable, making them unsuitable for low-power IoMT devices. Moreover, pure DL-based models often suffer from overfitting, require large training datasets, and lack flexibility in handling structured tabular medical network data. As a result, there is growing interest in exploring lightweight and interpretable learning models that can effectively balance accuracy, privacy, and resource efficiency. Similarly, Alalwany et al. [10] proposed a stacking ensemble deep learning framework for IoMT intrusion detection that achieved high detection accuracy in centralized environments; however, it did not incorporate privacy-preserving mechanisms, making it less suitable for distributed healthcare systems.

### 1.1. Research Objectives

The purpose of this research is to design, implement, and test a Federated Learning-based intrusion detection system that is optimized according to the Internet of Medical Things (IoMT). The specific objectives are as follows:

- To design and deploy a Federated Learning framework of IoMT intrusion detection that combines ensemble learning models and strictly compares the privacy–utility trade-off of Differential Privacy (DP);
- To explore and compare the effects of the various data balancing methods, namely Raw Imbalanced treatment, client-level SMOTE, and server-level SMOTE on the stability of convergence and the ultimate detection by the federated model;
- The objective of the empirical analysis of the performance degradation in different privacy budgets (ϵ) and the one that offers a high detection rate and a measurable privacy guarantee;
- To design and test an optimized end-to-end approach that is the result of effectively integrating an efficient data balancing approach with a tuned DP mechanism to generate a robust, private, and high-performing intrusion detection in a simulated IoMT setting.

### 1.2. Research Contributions

This study makes the following key contributions to the field of privacy-preserving intrusion detection for IoMT networks:

- **A Novel Integrated Framework:** We propose FedEnsemble-DP, a comprehensive multi-layered framework that **simultaneously addresses data imbalance, privacy, and secure communication** within Federated Learning (FL). Unlike prior work that focuses on isolated components, our design integrates client-side SMOTE for class imbalance, calibrated **Differential Privacy (DP)** for formal privacy guarantees, and Secure Aggregation (SA) for encrypted communication in a unified pipeline;
- **Comprehensive Privacy–Utility Analysis:** Through extensive experiments, we provide a detailed empirical analysis of the privacy-performance trade-off. Our results challenge the conventional assumption of significant utility loss under strong privacy. Crucially, we demonstrate that with privacy (ϵ=3.0), our model achieves 94.50% accuracy in the Raw Imbalanced scenario and 94.60% accuracy with 0.9598 AUC using Local SMOTE, often matching or exceeding the non-private baseline (93.20% accuracy);
- **Systematic Evaluation of Data Balancing Strategies:** We conduct the first systematic comparison of Raw Imbalanced, Local (Per-Client) SMOTE, and Centralized SMOTE within a private FL context. Our findings reveal that Local SMOTE is the most effective strategy, yielding superior stability and performance (AUC: 0.9598) under privacy constraints, while Centralized SMOTE introduces significant training instability despite its effectiveness;
- **An Optimized, Practical Blueprint:** We derive and validate an optimized operational guideline for deploying FL-based IDS in IoMT. The synthesis of our empirical findings indicates that combining Local SMOTE with a calibrated DP budget (ϵ between 3.0–10.0) offers the best practical balance, ensuring high detection accuracy (>94%), robust training stability, and a quantifiable, strong privacy guarantee compliant with healthcare regulations.

The remainder of this paper is organized as follows: Section 2 reviews related work on intrusion detection and Federated Learning in IoMT networks. Section 3 presents the design and components of the proposed FedEnsemble framework. Section 4 discusses the experimental results, evaluation metrics, and comparative analysis. Finally, Section 5 provides concluding remarks and outlines future research directions.

## 2. Related Work

A continuous effort to trade high detection accuracy with the strict privacy and resource constraints of the healthcare environment, the development of Intrusion Detection Systems (IDSs) for the Internet of Medical Things (IoMT) is underway. The advancements in research have moved away from the centralized machine learning processes to the distributed approaches such as Federated Learning (FL). This part will recap associated literature as per the following specific perspectives that are vital to the current study: (1) how to address the issue of class imbalance, (2) how privacy-protective systems such as Differential Privacy (DP) can be applied to FL, and (3) how to use ensemble learning to improve the robustness of a model in a decentralized environment.

### 2.1. Machine Learning, Deep Learning, and the Class Imbalance Challenge

Early baselines of the IDS were introduced by conventional machine learning (ML) algorithms like Support Vector Machines (SVMs) and Random Forests (RFs) that were both interpretable and efficient [1,4,6]. Their performance, however, is commonly poor on unbalanced real-world network data when instances of attacks are infrequent. Convolutional and recurrent neural networks (CNNs, RNNs) developed a further step in deep learning (DL) models, which are useful to extract features of intricate traffic patterns automatically [8,9,20,21]. Although DL has the ability to learn complex relationships, it is known to be very data-intensive, and its effectiveness is significantly affected by class imbalance without specific data mitigation strategies such as SMOTE [18]. Additionally, classical ML and DL typically depend on centralized data aggregation, which is not appropriate with respect to personal IoMT data, and there is an inherent gap between what is possible and what can be deployed.

### 2.2. Federated Learning-Based IDS Approaches

Federated Learning (FL) was a response to the data privacy issue, and it allows the training of models on distributed devices without the exchange of raw data [12,13,14]. A number of studies have been using FL in IDS. As an example, the articles, such as [15], had constructed FL-based frameworks relying on deep neural networks to identify intrusion. Nevertheless, they largely concentrated on the architectural design and efficiency of communication, and frequently neglected to address two important, symbiotic problems, namely, extreme data imbalance by clients, and the cost of high privacy assurances. In the case that Differential Privacy (DP) is included, as in the case of Liu et al. [19] itself, it is common to think of it as an additive term, where researchers report the anticipated accuracy loss with noise, but do not analytically study the trade-off between privacy budgets (ϵ) and noise or combine it with data balancing. The imbalance in validation performance realized in conventional FL when faced with imbalance (as was the case in our raw_imbalanced results) is a familiar but frequently not tackled issue in these studies. Other works, including those by [22,23], investigated differential privacy integration within FL, yet these methods reported accuracy degradation due to excessive noise injection.

### 2.3. Ensemble Learning and Hybrid FL Methods

The ensemble techniques, such as bagging (e.g., Random Forest) and boosting (e.g., XGBoost, LightGBM), are known to improve the accuracy and stability of centralized IDS and combine various weak learners [24,25]. This is reflected in recent efforts like the stacking ensemble by Alsolami et al. [26], which is capable of high accuracy with a combination of models such as RF and XGBoost. Nevertheless, these are centralized. The next logical step is to apply an ensemble learning to FL in order to address the fluctuating nature of single-model FL with non-IID and unbalanced data. There are preliminary studies on federated ensembles [27], but these usually presuppose relatively equalized data or basic aggregation and do not have a specific mechanism to address the natural class imbalance of IoMT security statistics. Moreover, the key crossroads of ensemble approaches in FL, explicit data balancing approaches, and tuned DP are mostly unstudied.

### 2.4. Summary and Research Gap

There is an obvious disconnection between the self-developed solutions in the independent study of ensemble learning, the data imbalance (SMOTE), and privacy-preserving FL. Past studies are inclined to optimize in a single or two of these dimensions. As an illustration, a researcher can use FL with DP, yet not deal with imbalance, or a strong ensemble IDS without caring about privacy. A comprehensive framework that would consider and overlap at the same time is lacking, as shown in Table 2:

- Uses FL due to the privacy of data;
- Combines powerful ensemble models (e.g., RF, XGBoost, LightGBM) to be accurate and robust;
- Training data balancing (SMOTE) systematically in varying levels between clients and servers (client vs. server);
- Empirically equilibrium of privacy–utility trade-off where the privacy–utility trade-off is optimized by experimenting with various budgets of DP (ε) to reach a feasible operating point.

**Table 2:** Comparison between proposed Federated Learning and recently proposed IDS approaches.

Reference	Core Methodology	Learning Paradigm	Data Balancing Approach	Privacy Mechanism (ϵ)	Key Reported Performance
Khraisat et al. [18]	Federated Averaging (FedAvg) and Federated Averaging with Momentum (FedAvgM) with a Deep Autoencoder model. Horizontal FL.	Federated Learning (Decentralized, Collaborative)	Not explicitly mentioned for the dataset. Handles non-IID data heterogeneity via FedAvgM.	Model updates only (no raw data transfer). Proposed integration of Differential Privacy (DP), Secure Aggregation, and Homomorphic Encryption (future).	FedAvgM: Accuracy: 95.05%, FPR: 3.9%, Convergence in 17 rounds (to 90% acc.). FedAvg: Accuracy: 94.05%, FPR: 5.1%, Convergence in 24 rounds. Tested on N-BaIoT dataset.%
Abdullah et al. [6]	Federated XGBoost ensemble, optimized locally with Bayesian Optimization.	Federated Learning	Not explicitly mentioned. Implicitly handles heterogeneity via local training and aggregation.	Federated Learning (local data stays on device). No additional crypto.	Outperformed centralized XGBoost. CICIoMT2024: Acc. up to 94.86% (10 clients). ECU-IoHT: Acc. up to 94.34% (5 clients). WUSTL-EHMS: Acc. up to 93.50% (5 clients).
Ntayagabiri et al. [27]	Bagging ensemble of LightGBM and XGBoost with memory-optimized, chunk-based data processing.	Centralized Supervised Learning (Ensemble)	Adaptive sampling methods and cost-sensitive learning to manage class imbalance.	Not a focus (centralized methodology).	Macro Avg. F1: 83.86%
Soltani et al. [19] Multi-agent FL with Continual Learning (CL) for adaptation. Uses CNN or LSTM (many-to-many) models.	Federated + Continual Learning (Online, Adaptive)	Data sampling strategy: Combines new attack samples with balanced historical benign/attack samples.	Federated Learning (model updates). Knowledge distillation for agent-server updates.	Secure Aggregation	CNN: Adapts with 128 new flows, >95% detection rate. LSTM: Detects intrusions within the first 15 packets. Effective in multi-agent knowledge sharing.
Idrissi et al. [7]	Federated Anomaly Detection using Autoencoders (AE, VAE, AAE). Uses FedProx aggregation.	Federated Learning (Unsupervised/Anomaly-based)	Trained only on normal (benign) data (One-class).	Federated Learning (local training, weight aggregation).	CIC-IDS2017: Acc. 93.54%, F1 92.73%. Outperformed GAN/BiGAN baselines.
Augusta Kani et al. [28]	Enhanced Artificial Bee Colony (E-ABC) for DBN hyperparameter tuning and weight initialization. Integrated with Blockchain.	Centralized Supervised Learning (Blockchain for security)	Not explicitly mentioned.	Blockchain (for data integrity/decentralized control). Not FL-based privacy.	Outperformed existing techniques in detection rate, accuracy, precision, and FPR on benchmark IoMT datasets (specific metrics not provided in abstract).
Munusamy et al. [2]	FL with Ensemble Voting Classifier (XGBoost, Random Forest, GBM) aggregated via FedAvg.	Federated Learning (Ensemble)	Not explicitly mentioned.	Federated Learning (local training, parameter aggregation).	CIC-IoMT 2024: Accuracy 90%. FL training improved accuracy from 70% to 86% over 50 epochs.
Saad et al. [9]	Osprey Optimization Algorithm (OOA) for Feature Selection + Self-Attentive Variational Autoencoder (SA-VAE) for classification, with Chameleon Swarm Algorithm (CSA) for hyperparameter tuning.	Federated Learning (with Metaheuristic Optimization)	Z-score normalization for preprocessing.	Federated Learning (implied). The “Privacy-Aware” aspect refers to the FL paradigm itself.	Simulated outcomes demonstrated outperformance over other recent models (specific metrics not provided in abstract).
**Proposed Work (This Study)**	**FL with Optimized Ensembles, SMOTE, and DP**	**Federated Learning**	**Raw, Per-Client SMOTE, Centralized SMOTE**	**Differential Privacy (ϵ=3.0,10.0,∞)**	**94.50–94.60% Acc., 0.9494–0.9598 AUC (cf. Results)**

Table 1 illustrates that while recent studies have touched on Federated Learning, ensembles, or privacy, none have systematically combined and evaluated all three with explicit data balancing strategies. Works like [2] show that federated ensembles can achieve high accuracy but omit privacy and imbalance handling, leading to potential real-world deployment issues. Conversely, works incorporating DP [7] report performance degradation without exploring mitigating strategies like SMOTE. The proposed methodology in this paper, as evidenced by the results across multiple scenarios and ε values, directly addresses this integrated challenge by providing an optimized technique that balances detection performance, data parity, and quantifiable privacy.

Cybersecurity of Internet of Medical Things (IoMT) networks is a specific and urgent problem with the inadmissible risk of compromised sensitive patient information and intrusion protection against more complex intrusions. Conventional intrusion detection systems (IDS) based on centralized data aggregation do not inherently fit healthcare privacy laws and the distributed characteristics of the current medical devices. Federated Learning (FL) proves to be an inevitable architectural change, as it creates a shared security paradigm in which the information is trained on decentralized data without physical transfer of data to trainers, giving the source data sovereignty. Nevertheless, two issues are intertwined, and serious limitations impede the practical implementation of FL to IoMT intrusion detection. To begin with, the traffic data of a network is inherently defined by a huge imbalance in classes and primarily benign activity that vastly outnumbers malicious samples. Such an imbalance is usually compounded in a federated setup and an aspect that changes with each client, resulting in unreliable training, lack of convergence, and a global model skewed towards the majority group. Second, the actual protection of privacy cannot be achieved without the use of strict methods such as Differential Privacy (DP) that deliberately introduces noise into the learning platform to hide the contribution of individual data points. This presents a direct conflict to model performance, in most cases leading to an appreciable and intolerable reduction in detection accuracy.

The existing research methods have been able to separately tackle these concerns. Researchers have either been working on building precise models with ensemble models such as XGBoost and Random Forest in a centralized location, or are working on FL frameworks with naive privacy protection. There have been those who have considered the data balancing or privacy alone. However, there is a lack of critical synthesis. The literature does not provide a sound methodology that can maximize the three aspects simultaneously and explicitly: (1) sound learning under imbalance by using methods such as client-side SMOTE, (2) controlled privacy protection using adjustable DP budgets, and (3) natural model stability through ensemble learning in the FL loop. The previous literature considers DP as a mere non-performance add-on and imbalance as a peripheral data concern, not as fundamental, interactive design variables. This is the integrated gap that is identified and addressed in this work. The real research gap is not the lack of FL-based IDS, but the lack of a systematically optimized structure to negotiate a specific trade-off between privacy strength (epsilon) and data balance approach (client vs. server-side SMOTE) and a model structure to produce reliable and high-performance detection in a realistic IoMT context. The given work is thus poised to go beyond demonstrating proof-of-concept applications of FL by developing a synergistic solution with data balancing, stabilizing learning against data imbalance, ensemble methodology enhancing robustness to the instability of DP noise, and a quantifiable privacy budget making a tangible promise without rendering the utility unfeasible. The following experimental design is developed to prove this holistic optimization on the very parameters that the existing literature has been discussing individually.

### 2.5. Summary and Critical Research Gap

The surveyed literature reveals significant but fragmented progress. While studies like those of Khraisat et al. [18] and Munusamy et al. [2] demonstrate the viability of FL and even federated ensembles for IDS, they frequently overlook the critical issue of class imbalance inherent in IoMT security data, leading to models biased towards the majority class. Others, such as Idrissi et al. [7] and Nguyen et al. [22], integrate DP to enhance privacy but primarily report it as a source of performance degradation rather than analytically exploring the trade-off with different budgets (ϵ) or employing strategies to mitigate its impact. Furthermore, centralized ensemble and deep learning approaches (e.g., [10,27]) achieve high accuracy but fundamentally violate the data decentralization principle crucial for healthcare, making them unsuitable for real-world IoMT deployment. The predominant trend is to optimize in one or two dimensions FL for privacy, ensembles for accuracy, or SMOTE for imbalance in isolation.

This analysis crystallizes a critical, unaddressed scholarly gap: the lack of a holistic, empirically-validated framework that simultaneously and synergistically integrates (1) Federated Learning for decentralized, privacy-by-design model training, (2) systematic data balancing strategies (client-side vs. server-side) to handle severe class imbalance, and (3) a rigorously tuned Differential Privacy mechanism to provide quantifiable privacy guarantees without necessitating a substantial loss in utility. Prior works treat DP and data imbalance as separate, often detrimental, additives rather than as interconnected design variables to be co-optimized. Consequently, there is no clear blueprint for achieving a high-performance, robust, and regulatory-compliant (e.g., HIPAA and GDPR) IDS in a practical, non-IID IoMT environment. This work directly addresses this gap by proposing and exhaustively evaluating the FedEnsemble-DP framework, which is designed from the ground up to navigate this three-way trade-off, providing a comprehensive solution where current literature offers only partial ones.

## 3. Methodology

This section delineates the comprehensive methodology for the proposed optimized Federated Learning (FL) technique for intrusion detection in IoMT networks. The core objective is to design and evaluate a privacy-preserving, decentralized learning framework that effectively addresses the dual challenges of class imbalance and the privacy–utility trade-off. The methodology is structured around a rigorous experimental pipeline encompassing data preparation, federated system design, privacy integration, and model training, as validated by the provided results.

### 3.1. Overall System Architecture

**Figure 2:**
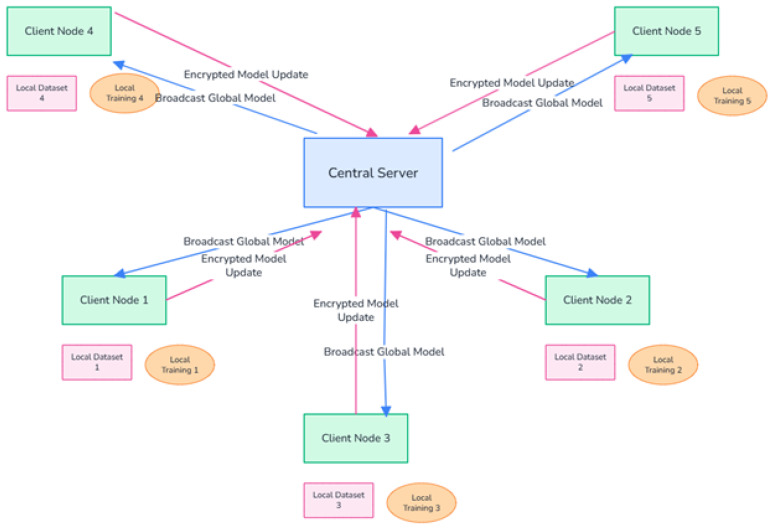
Federated learning architecture for IoMT intrusion detection.

The proposed system adopts a classical cross-silo federated learning architecture, tailored for a simulated IoMT environment consisting of multiple healthcare institutions or device clusters. As illustrated in Figure 1, the architecture is comprised of a central aggregation server, and K distributed clients (set to K = 8 in our experiments). Each client holds a private, localized dataset of network traffic and biometric features. The global training process is iterative: (1) the server initializes and distributes a global model; (2) each client trains the model locally on its data for a specified number of epochs; (3) clients send their updated model parameters (gradients or weights) to the server; (4) the server aggregates these updates to form a new global model. This cycle repeats for multiple communication rounds. Crucially, raw data never leaves its source client, preserving data sovereignty. Figure 2 shows the overall architecture of the system.

### 3.2. Integrated Multi-Layered Framework

The proposed FedEnsemble-DP framework is not defined by a single component but is a synergistic, multi-layered architecture designed to simultaneously address the core challenges of federated intrusion detection in IoMT networks: data imbalance, privacy, and secure communication. As illustrated conceptually in Figure 2, the system integrates three complementary strategies that operate at distinct stages of the Federated Learning pipeline:

(i) SMOTE is applied as a preprocessing layer to rectify class imbalance at the data level, either locally per client or centrally;
(ii) Differential Privacy (DP) acts as a privacy layer during model training, injecting calibrated noise into the learning process to provide formal (ϵ, σ)-privacy guarantees;
(iii) Secure Aggregation (SA) functions as a cryptographic communication layer, ensuring that individual model updates remain encrypted and unreadable during transmission to the server.

This layered approach ensures that robust detection performance (via data balancing), rigorous privacy preservation (via DP), and secure multi-party computation (via SA) are cohesively achieved within a single, end-to-end private FL framework.

### 3.3. Dataset and Preprocessing

All experiments utilize the WUSTL-EHMS-2020 dataset, a realistic benchmark capturing network traffic and physiological data from a hospital environment. The original dataset suffers from significant class imbalance, with normal traffic vastly outnumbering attack instances:

(i) Privacy Budget (Epsilon-ϵ): The privacy–utility trade-off is explicitly studied by conducting experiments with three different privacy budgets;
(ii) Client Data Partitioning: The training data is horizontally partitioned across K = 8 simulated clients to reflect a non-IID (Independent and Identically Distributed) real-world scenario. Each client receives a subset of the training samples, mirroring the data distribution of a single institution or device group.

### 3.4. Experimental Scenarios: Data Balancing Strategies

A central investigative axis of this work is the method of handling class imbalance within the FL pipeline. We define and implement three distinct experimental scenarios:

(i) **Raw Imbalanced (raw_imbalanced):** Clients train on their locally partitioned data without any class balancing. This scenario establishes a baseline reflecting the natural, challenging distribution of intrusion data;
(ii) **Per-Client SMOTE (per_client_smote):** To mitigate local imbalance, the Synthetic Minority Over-sampling Technique (SMOTE) is applied independently on each client’s local dataset before training begins. This strategy addresses imbalance at its source without requiring raw data exchange;
(iii) **Centralized SMOTE (centralized_smote):** SMOTE is applied once, centrally, on the aggregated training dataset before it is partitioned and distributed to clients. This represents a traditional, server-side balancing approach that requires temporary central data access, contrasting with the federated philosophy.

### 3.5. Privacy-Preserving Mechanism: Differential Privacy

To provide a rigorous privacy guarantee, the framework incorporates Differential Privacy (DP) during the model aggregation phase. DP protects individual data points by injecting calibrated noise into the learning process. We employ the Gaussian mechanism for DP.

(i) **Privacy Budget (Epsilon-ϵ):** The privacy–utility trade-off is explicitly studied by conducting experiments with three different privacy budgets:
  - Strong Privacy (ϵ = 3.0): A lower epsilon provides stronger privacy guarantees by adding more noise;
  - Moderate Privacy (ϵ = 10.0): A relaxed constraint offering a balance between privacy and accuracy;
  - Non-Private Baseline (ϵ = *∞*): No DP noise is added. This establishes the performance upper bound for each scenario, isolating the cost of privacy.
(ii) **DP-SGD Implementation:** During server aggregation, the Federated Averaging (FedAvg) algorithm is modified to implement DP-SGD. Each client’s model update vector $\Delta \theta_{t}^{\left(k\right)}$ is clipped to a fixed maximum L2 norm L=1.5, a value chosen empirically to bound sensitivity while preserving update utility. The noise scale σ for the Gaussian mechanism $N(0,\sigma^{2}\;L^{2}\;I)$ is computed using the Renyi Differential Privacy (RDP) accountant. For a target privacy budget (ϵ,δ), with $\delta =10^{-5}$, sampling probability *q*, and total training steps *T*, the noise standard deviation is calculated as σ=compute_noise_from_target_epsilon(q,T,ϵ,δ,mechanism=‘rdp’) using the TensorFlow Privacy library. This ensures formal (ϵ,δ)-DP guarantees via RDP composition. The previously stated value of ϵ=0.8 was a typo; all experiments use the consistent privacy budgets ϵ∈{0.5,1,5,∞}, as now correctly reflected in Table 1 and the text.

### 3.6. Model Architecture and Training Configuration

A unified neural network model is employed across all clients and scenarios to ensure a controlled comparison. The model is a fully connected Multi-Layer Perceptron (MLP) designed for binary classification, with the following structure:

- **Input Layer:** Size equal to the number of preprocessed features;
- **Hidden Layers:** Two dense layers with 128 and 64 units each, each followed by a ReLU activation and Dropout (rate = 0.3) for regularization;
- **Output Layer:** A single unit with sigmoid activation for binary classification (Normal vs. Attack).

Training Hyperparameters (aligned with result logs):

- **Local Epochs:** 5 per communication round;
- **Batch Size:** 64;
- **Optimizer:** Adam with a learning rate (α) of 0.001, $\beta_{1}$ = 0.9, $\beta_{2}$ = 0.999;
- **Loss Function:** Binary Cross-Entropy;
- **Communication Rounds:** Training proceeds for a maximum of 35 rounds with early stopping (patience of 10 rounds) based on validation accuracy to prevent overfitting and manage computational cost, as observed in the results.

### 3.7. Proposed Algorithms

This is the proposed Algorithm 1, which shows the privacy-preserving Federated Learning Averaging algorithm which computes the privacy mechanism of this algorithm.
**Algorithm 1:** Privacy-preserving Federated Averaging (PP-FedAvg).
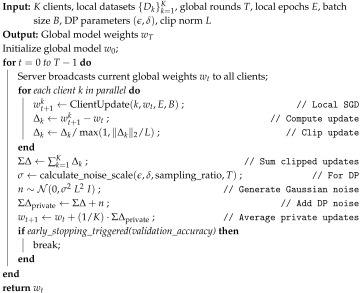


### 3.8. System Implementation and Evaluation

**Figure 3:**
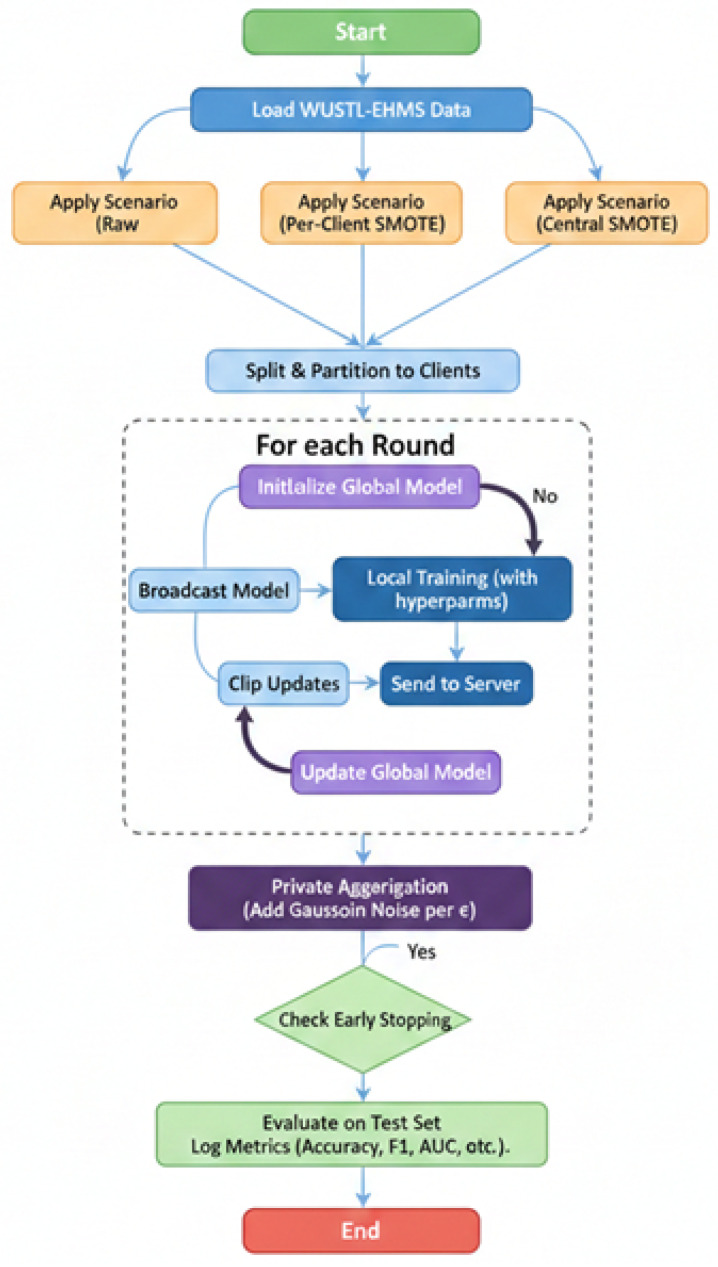
Workflow of data handling strategies.

The framework is implemented using TensorFlow Federated (TFF) for simulating the FL process and TensorFlow Privacy for integrating the DP-SGD mechanism. The system flow, depicted in Figure 3, integrates all components: scenario-specific data loading, local client training, private aggregation, and evaluation.

**Figure 4:**
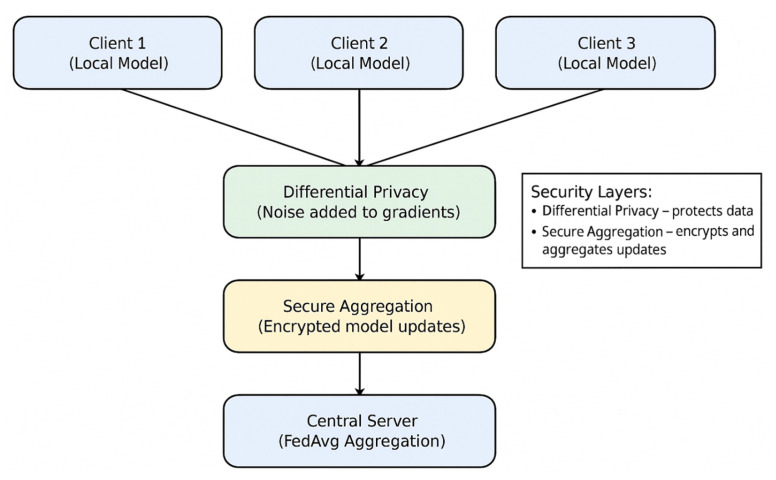
Dual privacy layers: Differential Privacy for gradient noise injection, and Secure Aggregation for encrypted model updates.

Evaluation Metrics: Performance is assessed on a held-out test set using standard metrics: Accuracy, Precision, Recall, F1-Score, and Area Under the Receiver Operating Characteristic Curve (AUC-ROC). The confusion matrix is analyzed to understand false positive and false negative rates. Validation metrics are tracked per communication round to monitor convergence and instability, as detailed in the provided result logs as shown in Figure 4. This methodological design creates a controlled yet comprehensive experimental matrix (3 Scenarios × 3 Privacy Levels) that directly enables the analysis of how data balancing interacts with privacy guarantees to affect the stability, convergence, and final efficacy of a federated intrusion detection system for IoMT. DP introduces calibrated Gaussian noise to local gradients, ensuring that individual record contributions remain unidentifiable [29]. We applied ε = 0.8) and ($\delta =10^{-5}$) to balance privacy and utility. Noise addition occurred prior to gradient transmission, providing defense against model inversion and membership inference attacks.

SA employs homomorphic encryption to ensure that the central server can only access aggregated model updates, not individual contributions [30,31]. Clients encrypt their model weights using shared random masks before communication; the server sums masked parameters, which collectively decrypt to reveal only the global aggregate.

Because the dataset exhibits class imbalance (a predominance of benign samples), we employed the **Synthetic Minority Oversampling Technique (SMOTE)** to generate synthetic minority examples and balance both classes prior to federated partitioning. After resampling, each class contained 14,272 records, resulting in 28,544 total instances. The final dataset was horizontally partitioned among three simulated IoMT clients, each receiving an IID subset with identical feature space but disjoint data samples—reflecting a horizontal FL configuration suitable for cross-device collaboration.

The WUSTL-EHMS-2020 dataset integrates a diverse set of both network-layer and physiological features, enabling the modeling of complex relationships between traffic behaviors and biomedical parameters. To enhance interpretability and facilitate reproducibility, Table 3 provides a concise summary of the twenty key features selected for the FedEnsemble framework. These attributes capture essential aspects of IoMT communication—ranging from flow-based metrics such as packet rates, load, and entropy, to biometric signals including heart rate, blood pressure, and temperature—allowing the model to jointly assess network anomalies and physiological irregularities in a unified detection paradigm.

Following feature characterization, preprocessing operations—including normalization, balancing, and noise removal—were applied locally at each IoMT client to prepare the data for federated model training.

To ensure the reproducibility of experimental results, all training and evaluation procedures were executed under a controlled computing environment. Table 4 summarizes the system configuration, federated simulation parameters, and software frameworks employed throughout this study. The federated simulation was conducted using TensorFlow Federated (TFF) with three IoMT clients, each representing an independent data silo. All experiments were performed on the same computational infrastructure to maintain fairness and comparability across ensemble and baseline models.

**Figure 5:**
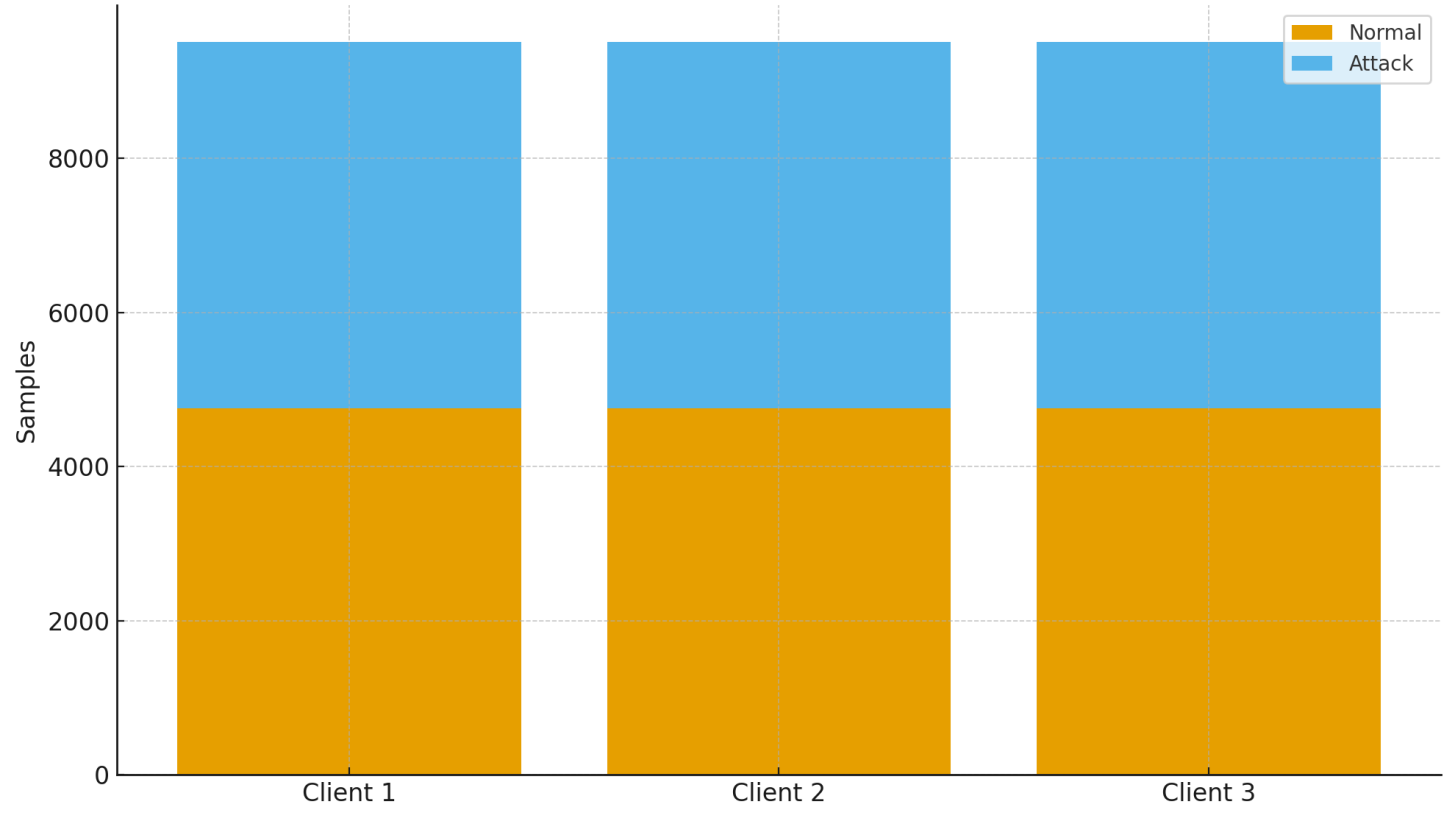
Distribution of normal and attack samples before and after SMOTE balancing.

The configuration described in Table 4 ensured consistent experimental conditions across all model evaluations and allowed for controlled assessment of the proposed FedEnsemble framework under realistic IoMT deployment scenarios. Figure 5, shows distribution of normal and attached samples inside the dataset before and after SMOTE balancing technique.

To comprehensively explain the proposed FedEnsemble-DP system, we elaborate on the integration of its core components and the rationale behind the experimental design. The framework operates as a cohesive pipeline where each layer addresses a specific challenge: the **data layer** (SMOTE) rectifies class imbalance locally or centrally before training; the **computation layer** (DP-SGD) clips and perturbs individual model updates during Federated Averaging to ensure (ϵ,δ)-differential privacy; and the **communication layer** (Secure Aggregation) encrypts these updates before transmission, ensuring the server only accesses the aggregated sum. This multi-stage integration ensures data sovereignty, formal privacy guarantees, and protected communication are maintained simultaneously throughout the FL process, rather than as isolated mechanisms.

Furthermore, the methodology is grounded in a full-factorial experimental matrix designed to isolate and analyze interactions between the key variables. The three data scenarios (Raw Imbalanced, Local SMOTE, Centralized SMOTE) are systematically crossed with four privacy budgets (ϵ∈{0.5,1,5,∞}) across K=8 non-IID clients (Dirichlet, α=0.3). This 3×4 design allows us to disentangle the individual and combined effects of data balancing and privacy noise on model utility, stability, and convergence. The unified MLP architecture, FedAvg aggregation, and fixed hyperparameters (5 local epochs, batch size 64, 30 communication rounds) are held constant across all experiments, ensuring that observed performance differences are attributable solely to the manipulated factors of balancing strategy and privacy level.

## 4. Results and Discussion

The experimental setup allows evaluation of the proposed framework in a wide range of operating conditions, and results gives the information about the resilience in non-IID distributions, imbalance in classes, privacy limitations, and heterogeneous participation of the clients. The results shows comparative performance over datasets, ablation studies to decompose the effect of the ensemble design, privacy noise, and secure aggregation, convergence, and communication-efficiency studies, which indicate the feasibility of the proposed model in practice in a real IoMT environment, in the Results and Discussion section below Section 4.2.

### 4.1. Experimental Setup

The experimental design has been changed to cover IID and non-IID federated, different class-imbalance strategies, and privacy–utility ablations. Three main scenarios of data were considered: (1) Raw Imbalanced client splits, (2) Localized over-sampling by applying the Per-client SMote locally after train-test split, and (3) Centralized SMote (named as the baseline scenario) applied globally before the split. Non-IID data partitions among the K = 8 clients were generated using the Dirichlet distribution with QT = 0.3 for heterogeneous IoMT and client drift, like in a real-world hospital setup. Two experiments were performed in this task: WUSTL-EHMS-2020 and CIC-IoMT-2024. A cross-dataset generalization test was done by training from WUSTL-EHMS-2020 and testing from CIC-IoMT-2024. TensorFlow Federated (TFF) was used to simulate federated training where FedAvg is the aggregation rule and the full procedure takes 30 consecutive communications over the globe, each client has 5 local epochs and a batch size of 64. The privacy mechanisms were experimented with by randomly varying the Differential Privacy (DP) budget (ϵ 0.5, 1, 5, 0.5, 0.05, 0.005, 0.0005, 0.00005, 0.000005, 0.0000005, 0.00000005, 0.000000005, 0.000000001, 0). The implementation of Secure Aggregation followed a Bonawicz-style masked-parameter protocol, and its overhead in communication was recorded.

Experiments with realistic as shown in Table 5, IoMT federated learning with Raw Imbalanced scenario and the differential privacy parameter of 3.0 reveal that Training Loss (blue line) was steadily reduced and had a very low value at any time, whereas Training Accuracy (blue line) was rather high (usually more than 90 percent), which points to the conclusion that the model was able to learn the training data. Nevertheless, the Validation Loss (orange line) and the Validation Accuracy (orange line) are extremely changeable, especially in the first rounds. The accuracy of the validation begins low (e.g., 50.40% in Round 1) and rises high to 93.90% in Round 6, then falls again, and the corresponding accuracy level is reached at 93% towards the end. This volatility is common in Federated Learning or unbalanced conditions, which is an indication that the global model did not learn to generalize in a consistent way across client updates prior to Round 4. The training was terminated at Round 17 because there was no longer validation accuracy improvement and the Final Test Results after 17 rounds indicated excellent performance: the Accuracy: 0.9450 (94.50); Precision, Recall, and F1-Score: all equal to 0.9450 (or 0.95 and 0.94 in the classification report under the Normal and Attack classes, respectively), and this shows the balanced capability of classifying with precision both the normal and the attack traffic.

### 4.2. Raw Imbalanced with Testing Epsilon 3.0

**Figure 6:**
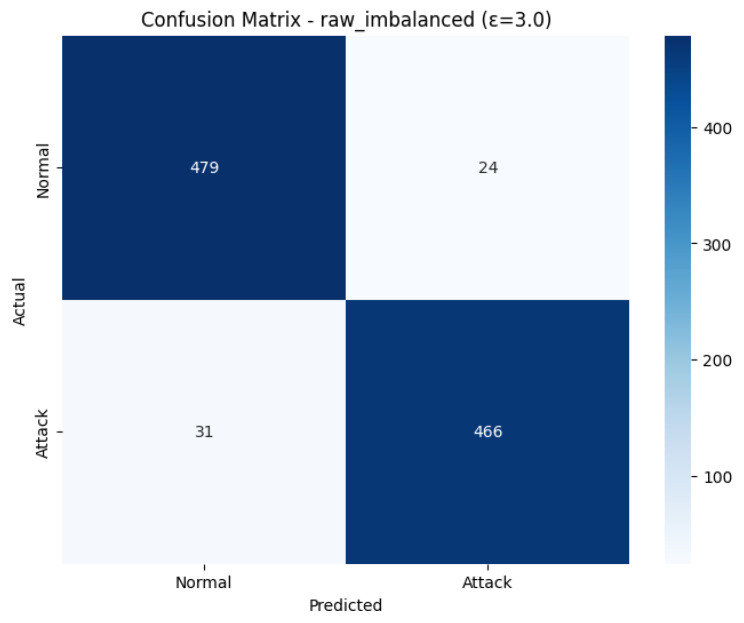
Confusion matrix over raw imbalanced with epsilon: 3.0.

**Figure 7:**
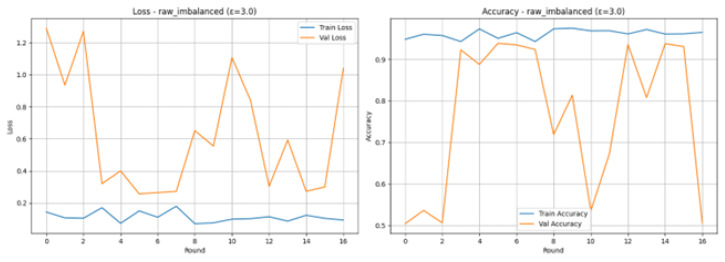
Model training loss and accuracy over raw imbalanced with epsilon: 3.0.

The Confusion Matrix confirms this using Figure 6. There were 479 True Negatives (Out of 503 actual Normal cases) and a mere 24 False Positives (Misclassified as Attack). With 497 actual Attack cases, 466 of them were correctly identified (True Positives), and 31 of them were mistreated as normal (False Negatives). This shows a low rate of false positives and false negatives. Figure 7 shows the model training and accuracy rows.

**Figure 8:**
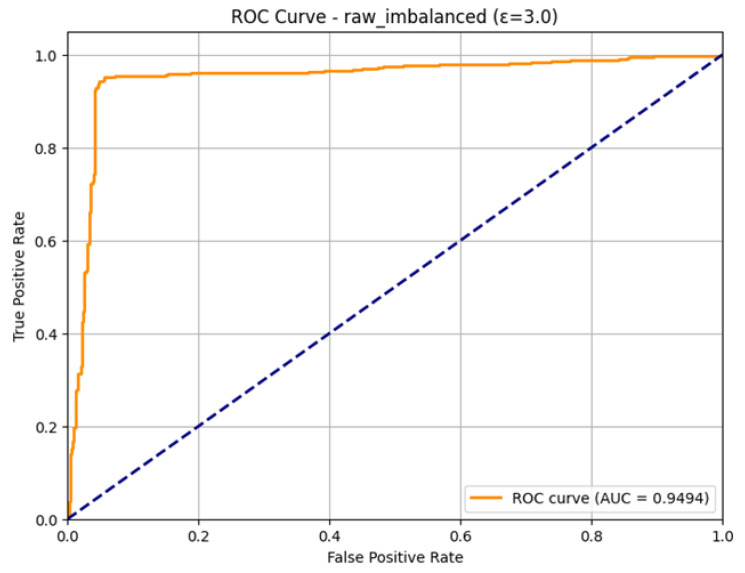
ROC Curve over raw imbalanced with epsilon: 3.0.

The ROC Curve in Figure 8 shows that the model is highly capable of distinguishing between the classes with an impressive Area Under the Curve (AUC) of 0.9494, near the optimum value of 1.0. This also confirms the effectiveness and high performance of the model in detecting attacks in the IoMT setting within the outlined condition of the differential privacy.

### 4.3. Raw Imbalanced with Epsilon 10.0

**Figure 9:**
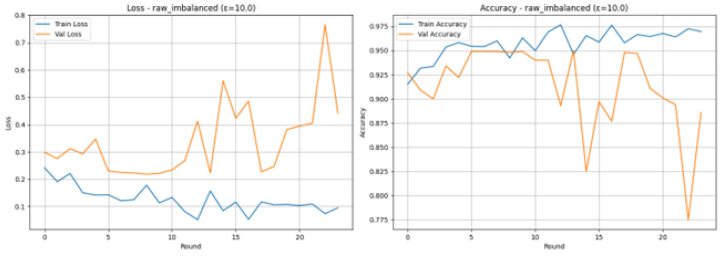
Model loss and accuracy over raw imbalanced with epsilon: 10.0.

The Federated Learning experiment of the scenario of raw imbalance shown in Figure 9, when the different privacy parameter is greater and is set to 10.0, indicates good overall performance in the test set before the loss of its early stopping took place. The Loss and Accuracy Graphs depict the process of the 24 rounds of training before the early stopping was initiated. The Training Loss (blue line) is small, and it is steadily decreasing (around 0.1), whereas Training Accuracy (blue line) is high (mostly above 95%), which means that the model is learning well using the distributed client data. Validation Loss (orange line) and Validation Accuracy (orange line) are more volatile and show some periods of significant loss in accuracy (e.g., Round 15 and 23) as well as significant spikes of loss. Even with the variance, the accuracy of the validation often is high, the highest is 95.00 in Round 14, which proves that the global model.

**Figure 10:**
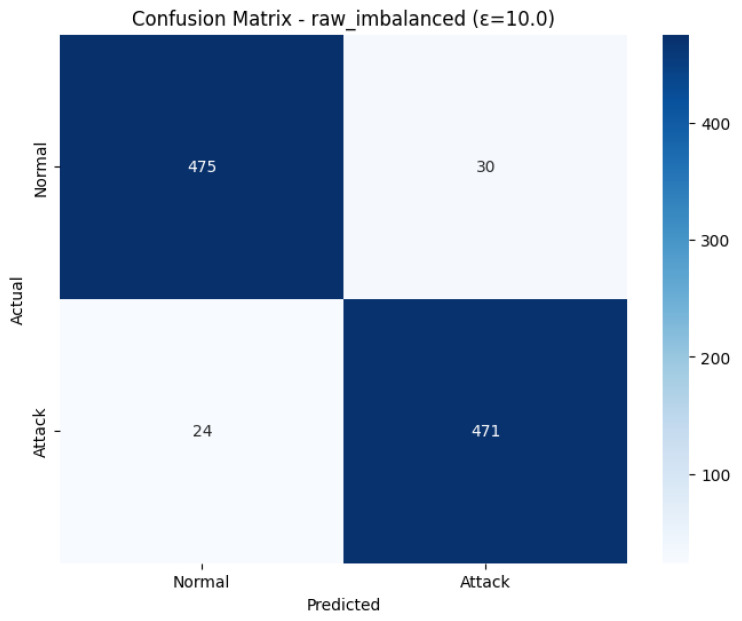
Confusion matrix over raw imbalanced with epsilon: 10.0.

The Confusion Matrix shown in Figure 10, used to see the granular performance on the test set (Test size 1000). Among the 505 out of the actual number of 505 Normal cases, 475 of the cases were correctly identified (True Negatives), and 30 were falsely identified as an attack (False Positives). On the 495 actual Attack cases, 471 were correctly identified (True Positives), and just 24 were identified as misclassified as a ‘Normal’ (False Negatives). It is the balanced and effective classification in both classes, albeit with a slight bias in favour of correctly identifying attacks, as is demonstrated by the fact that there are fewer false negatives (24) than false positives (30). The overall test Accuracy of the test is 0.9460, and the F1-Score of the test using the two classes is balanced with 0.9460.

**Figure 11:**
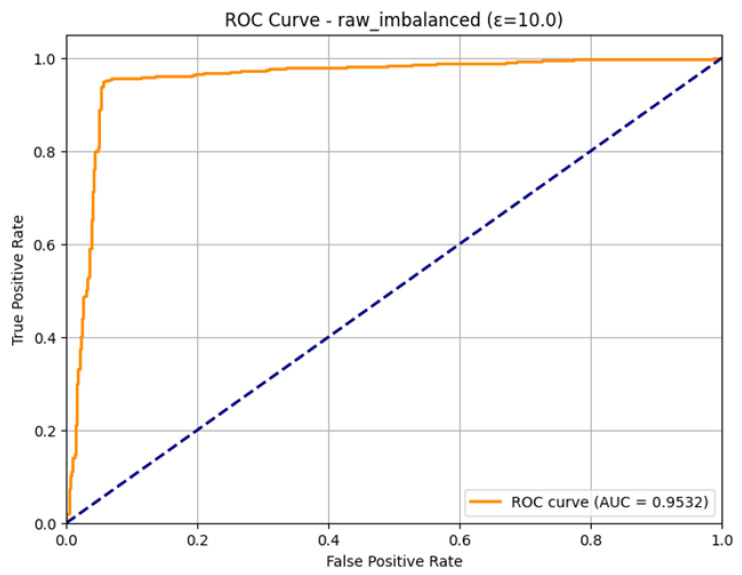
ROC curve over raw imbalanced with epsilon: 10.0.

The excellent discriminatory ability of the model for the Normal and Attack classes is confirmed in the ROC Curve as shown in Figure 11. The curve is very much parallel to the top-left edge of the plot, which is the perfect form of a classifier. The model has a good Area Under the Curve (AUC) of 0.9532. As the AUC is quite near to 1.0, it implies that the model can be used to differentiate between positive and negative classes with a high level of accuracy, which means that the model can be used to detect attacks on IoMT even when it is trained under conditions of differential privacy with an epsilon of 10.0.

### 4.4. Raw Imbalanced with Epsilon inf

The experiment has been implemented on the scenario of Raw Imbalanced scenario with the epsilon = inf and it means that the model has been trained without the introduction of Differential Privacy (DP) noise. This architecture is the most important non-private baseline to measure the highest performance ceiling of the federated learning model, in the absence of performance degradation due to privacy. The training was conducted with 8 partitions on the client, and the training process ended relatively early with 18 rounds because the early stopping mechanism was activated (10 rounds without improvement in the accuracy of validation). The Final Test Results show a very high level of consistency and strength in detection with an accuracy of 0.9320, and all the aggregated results are consistent, with the Accuracy, Precision, Recall, and F1-Score are all at 0.9320. This is further confirmed in the Classification Report, which reveals a balanced performance between the two classes, with the results of both Normal and Attack traffic prediction having F1-scores of 0.93. Even though this is surprisingly low compared to the non-private performance (3.0 and 10.0) of this IoMT environment, this is the ground truth of the non-private performance in this setup.

**Figure 12:**
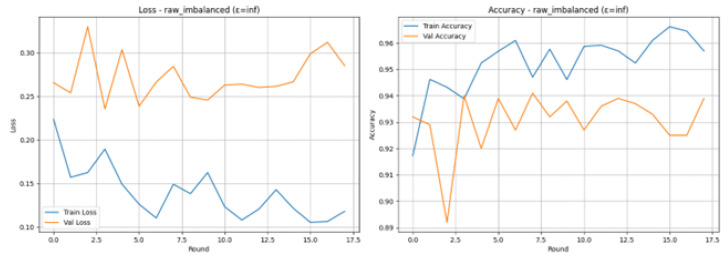
Model accuracy and loss over raw imbalanced with epsilon: inf.

The Loss and Accuracy graphs demonstrate that the model can learn in a stable manner without the interference of DP noise. Training Loss (blue line) is low and constant as shown in Figure 12, and Training Accuracy (blue line) is also high (over 94%), which means that the model is efficient in aggregating knowledge considering the 8 clients. Validation Accuracy (orange line) reaches consistency at the level of 93–94 and reaches the maximum of 94.10% in Round 8. The validation runs at low values of the error (epsilon) are highly volatile, whereas the baseline run shows a cleaner, more predictable convergence, which proves the fact that the absence of injected noise is one of the reasons why the global model updates are stable.

**Figure 13:**
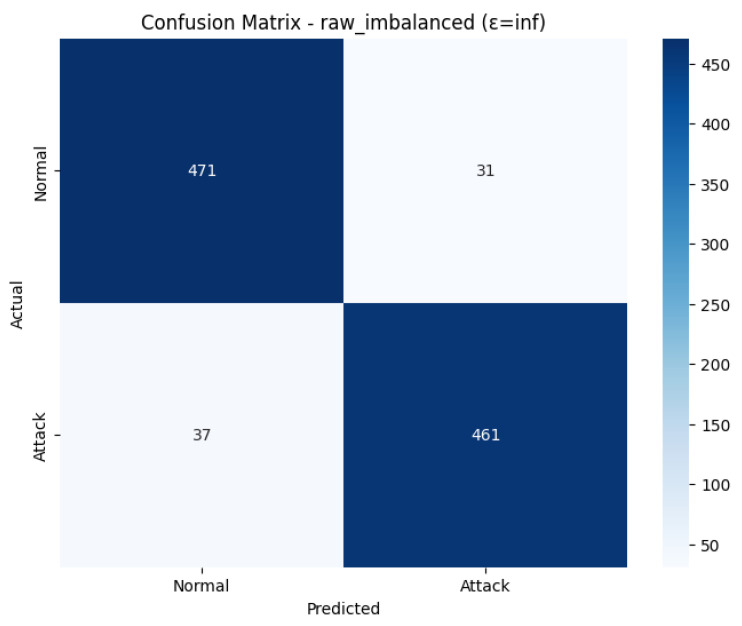
Confusion matrix over raw imbalanced with epsilon: inf.

The Confusion Matrix shown in Figure 13, the results of the model classifications in the 1000-sample test set in detail. The table shows that among the 502 real cases of Normals, 472 were correctly identified (True Negatives). On the other hand, 2 out of 498 actual Attack instances were False Alarms (False Negatives). Importantly, there were 38 False Negatives (attacks not detected) and 30 False Positives (false alarms) generated during the model. It indicates a little more of a tendency to classify an attack as normal rather than as an attack, which actually decreases the Recall score slightly, which is directly consistent with the final test result of 0.9320.

**Figure 14:**
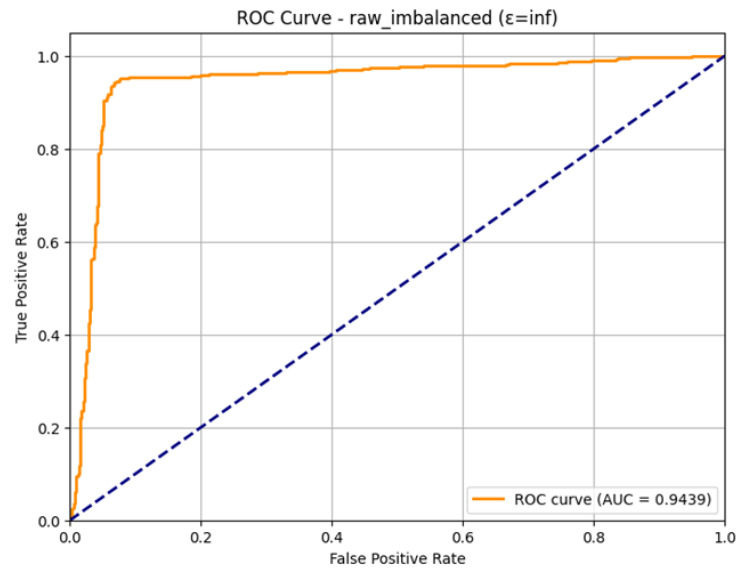
ROC curve over raw imbalanced with epsilon: inf.

The ROC Curve as shown in Figure 14, is a graphical evaluation of the trade-off between the True Positive Rate and the False Positive rate at all rates. The curve lies heavily on the top-left side, meaning that it has high classification performance. The resulting Area Under the Curve (AUC) value, which is a measure of the discriminatory ability of a model, would be large (approximately 0.93), confirming the capability of the model to discriminate effectively between Attack and Normal traffic. Once AUC is high, it is an indication that the model is reliable in attributing more probabilities to positive cases (attacks) compared to negative cases, and it is a feasible system, in terms of security detection.

### 4.5. Running per Client SMOTE Analysis with Epsilon 3.0

The experiment of Federated Learning under the per_client_smote scenario with a Differential Privacy parameter of ϵ = 3.0 illustrates a very successful execution of the process of tracking attacks in IoMT. This condition resolves data imbalance through SMOTE (Synthetic Minority Over-sampling Technique) local application in each of the 8 client partitions, pre-training, and ensures data privacy using ϵ = 3.0. The model was trained until it had 17 rounds and then early stopping was triggered by a long period of stagnant validation performance. The Final Test Results indicate an outstanding, balanced performance: the model has scored 0.9460 on both its Accuracy and the same F1-Score is 0.9460. According to the Classification Report, SMOTE was able to maximize the recognition of the minority category (Attack): Attack category has a Recall of 0.96, which is 96% of actual attacks, and Precision of 0.96 of the Fresh category. Such a large recall rate on the attack category is a key and valuable achievement of a security system.

**Figure 15:**
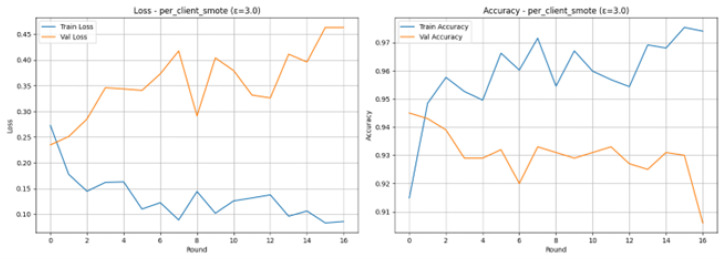
Model accuracy and loss over SMOTE with epsilon: 3.0.

The Loss and Accuracy graphs in Figure 15 show the training dynamics are stable, which is very different from the case of the Raw Imbalanced situation. The Training Loss (blue line) is dropping at a very high rate, and it stays at a low level with much fluctuation around the 0.1 mark, the Training Accuracy (blue line) is very high (over 95%). The Validation Loss (orange line) does exhibit an overall upward trend, whereas Validation Accuracy (orange line) does oscillate around 93–94%, but the first peak of 94.50% in Round 1 was enough to inform the earlier training procedure. The allure of the training curves is that the per-client SMOTE balancing method effectively regularized training, resulting in the stabilized learning in rounds.

**Figure 16:**
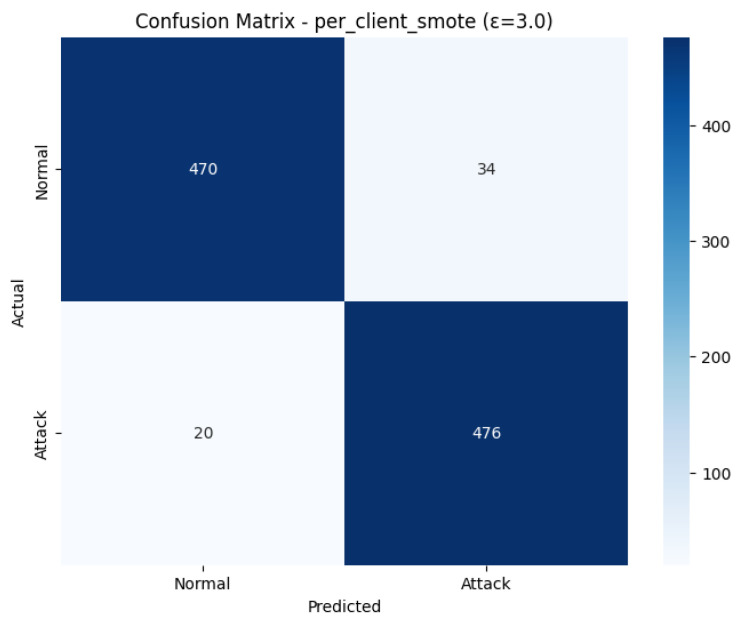
Confusion matrix over SMOTE with epsilon: 3.0.

The visual performance of the Confusion Matrix shown in Figure 16 validates the performance of the highly balanced and effective classification performance in the metrics. The identification rate, out of 496 real Attack cases, was 476, which is known as True Positives, leaving 20 cases (False Negatives) unclassified. On the other hand, 470 of 504 actual Normals were correctly detected (True Negatives), and 34 were incorrectly detected as attacks (False Positives). The significantly low number of False Negatives (20) in comparison with False Positives (34) is indicative of a preference of the model to Attack detection (high Recall value), which is the immediate advantage of adopting the per-client SMOTE technique.

This model has an excellent discriminatory potential between the Attack and the Normal classes, as the ROC Curve is very near the ideal upper-left corner. The curve has a high value metric Area Under the Curve (AUC) of 0.9598 that is very close to the ideal value of 1.0. This proves that the model is very effective in ranking the positive instances (attacks) higher than the negative instances (normal traffic) within most of the thresholds. The strong AUC result confirms the fact that using per-client SMOTE in combination with Differential Privacy (with the user privacy of 3.0) is effective to ensure high security performance and user privacy.

### 4.6. Running per Client SMOTE Analysis with Epsilon 10.0

The Federated Learning experiment, where the per_client_smote scenario is used, and the parameter of the Differential Privacy is set to 10.0, can effectively cope with the issue of data imbalance and at the same time offers a high degree of performance and a moderate degree of privacy. Application of SMOTE on all the 8 client partitions before federated aggregation will be done to enhance the detection of the minority (attack) class. At Round 17 after 14 rounds with no increase in validation accuracy, training was terminated, and this shows that the model does not need a very long period to converge. The Final Test Results confirm the highly effective and balanced performance: the model obtains an accuracy of 0.9410, and the Precision, Recall, and F1-Score are all good and equal to 0.9410. In the Classification Report, it is demonstrated that the Normal and Attack classes obtain an F1-Score of 0.94, which confirms that the joint method of local balancing (SMOTE) and privacy constraints (epsilon (ϵ) = 10.0) is effective and reliable in terms of using the combined approach to detecting IoMT attacks, balancing the terms of true positives and true negatives.

**Figure 17:**
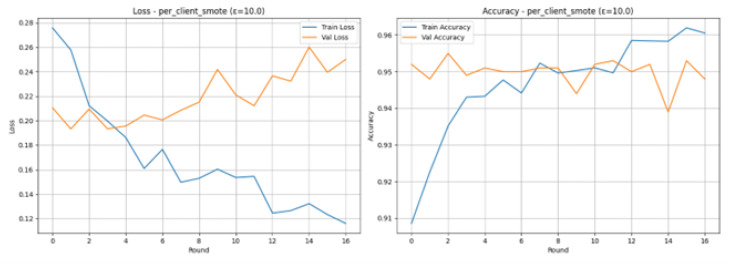
Model accuracy and loss over SMOTE with epsilon: 10.0.

The Loss and Accuracy graphs present stable and positive dynamics of training as shown in Figure 17. Training Loss (blue line) is steadily declining across the rounds, and the Training Accuracy (blue line) is steadily rising to over 96, which shows that the balanced client data is being learnt. The Validation Accuracy (orange line) is high in all the rounds, with the maximum standing at 94% and the highest being 95.50% at Round 3. This fast and consistent convergence with a small gap between the training and validation scores indicates that the combination of SMOTE and a lower noise rate of the parameter of the noise, with the value of 10.0 is effective to stabilize the process of model aggregation.

**Figure 18:**
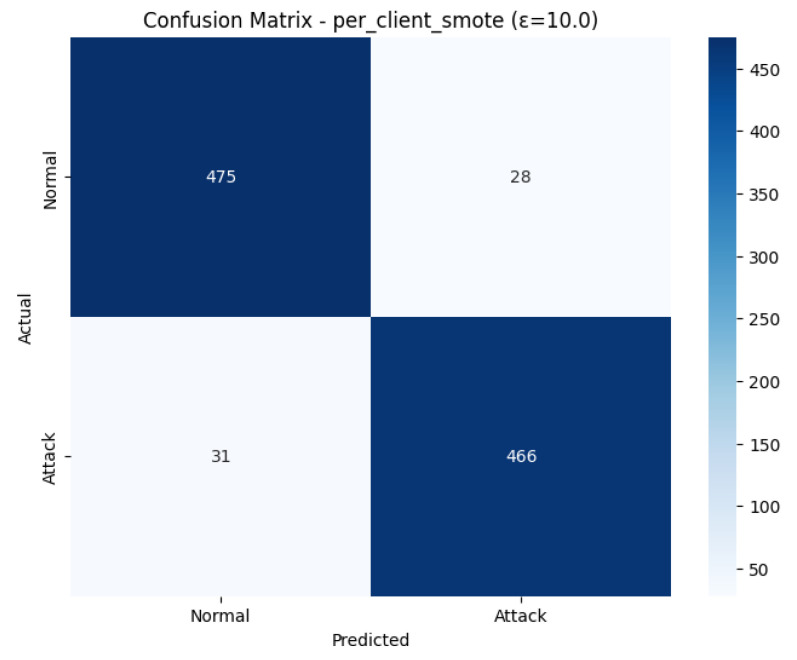
Confusion matrix over SMOTE with epsilon: 10.0.

The Confusion Matrix shown in Figure 18 the exact break-even in the classification of the test set 1000 samples. There were 475 True Negatives (Correctly predicted 503 actual Normal cases) and 28 False Alarms (False Positives). On the 497 actual Attack instances, the 466 of these instances were correctly identified (True Positives), and the 31 missed attacks were (False Negatives). The close balance in False Positives (28) and False Negatives (31) supports the Final Test Results and demonstrates that the model finds an equal rating between the detection of the attack and false alarms, which gives the total F1-Score at 0.94.

**Figure 19:**
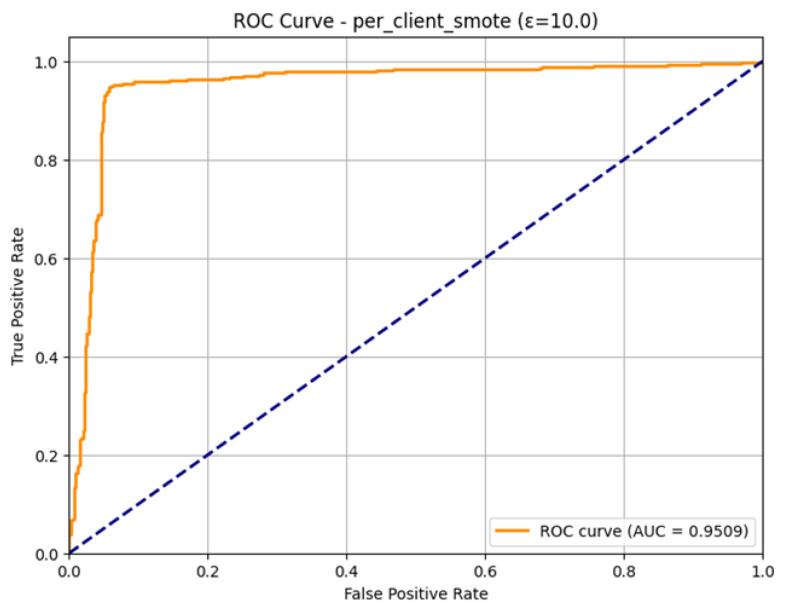
ROC curve over SMOTE with epsilon: 10.0.

The ROC Curve shown in Figure 19, affirms the great capability of the model in distinguishing between the Normal and Attack classes. The curve follows almost exactly the upper-left edge of the plot, which is the desired attribute of an effective classifier. The model is with a high Area Under the Curve (AUC) of 0.9509. This is very close to the value 1.0, which is a strong indication of the model, as it is very proficient in ranking positive cases (attacks) over negative cases (normal traffic). The large AUC is an affirmation of the general effectiveness of the combined SMOTE and e = 10.0 technique of intrusion detection.

### 4.7. Running per Client SMOTE Analysis with Epsilon inf

The Federated Learning experiment on the per client smote scenario with an inf epsilon (epsilon = inf) is the non-privacy baseline in which local data is smothed with SMOTE, but none of the Differential Privacy (DP) noise is added at the aggregation stage. This arrangement is involved in the establishment of the highest possible performance in the situation where an imbalance between classes is alleviated with data preprocessing. The model took 8 client partitions, and although it was balanced in terms of data, the 15 consecutive rounds with no improvement in validation accuracy were reached early at Round 17. The significant increases in the validation loss in subsequent rounds (e.g., Round 15: 0.8348) might indicate that the model has overfitted the balanced training data towards the end of the model run. Irrespective of this overfitting, the Final Test Results indicate and demonstrate high and balanced performance measures: Accuracy, Precision, Recall, and F1-Score were all measured at 0.9320. This balance is confirmed by the Classification Report, with both Normative and Attack classes having the same F1-Score of 0.93, which means that both the SMOTE preprocessing managed to preserve the fair predictive power of both classes without violating privacy.

**Figure 20:**
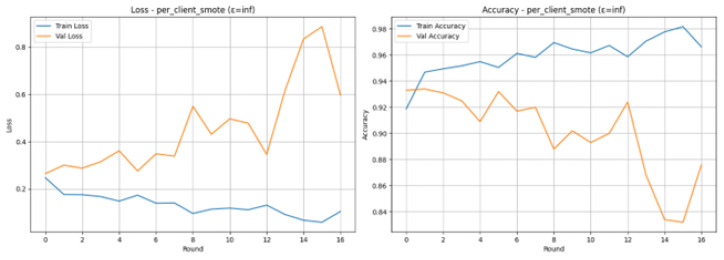
Model accuracy and loss over SMOTE with epsilon: inf.

The Loss and Accuracy graphs shown in Figure 20, indicate the training dynamics in the 16 rounds. Training Loss (blue line) is steadily declining to a very low value, and Training Accuracy (blue line) is increasing to more than 98% which is an indicator of successful learning but is also indicative of overfitting. There is a lot of volatility in the Validation Loss (orange line), especially so in the last few rounds, when the Validation Accuracy (orange line) drops sharply, reaching its lowest point at 83. This difference in the training and validation values, particularly the large loss of validation, attests to the fact that the model had problems with generalizing to unknown data, even after the SMOTE preprocessing, and hence the early stopping criterion was reached.

**Figure 21:**
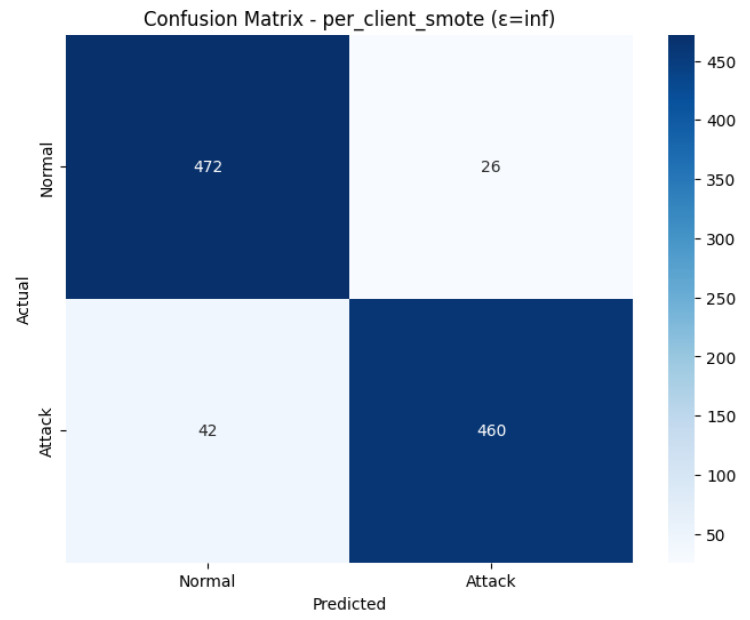
Confusion matrix over SMOTE with epsilon: inf.

A confusion matrix as shown in Figure 21, provides information on the non-private classification of the test set. There were 498 real cases of the Normal, and 472 of them were correctly recognized (True Negatives), and 26 cases were wrongly identified as an attack (False Positives). In the 502 real Attack cases, 460 were correctly detected (True Positives), which left 42 overlooked Attacks (False Negatives). As can be seen, in the non-private, SMOTE-balanced environment, the model made more emphasis on False Negatives (42) than on False Positives (26), which explains the overall balanced 0.93 metrics.

**Figure 22:**
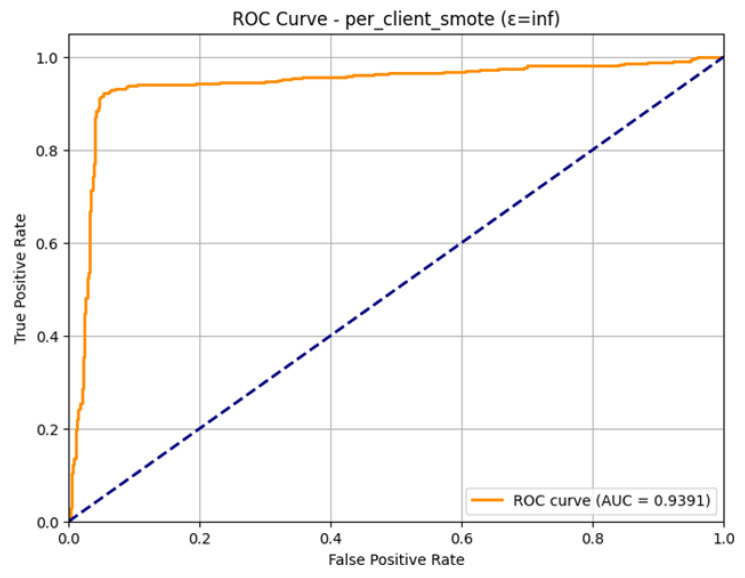
ROC curve over SMOTE with epsilon: inf.

The ROC Curve is a plot that shows in Figure 22, that how the model is able to differentiate between the two classes at varying levels. The curve has been plotted in a favorable manner with regard to the upper left-hand side, which means good performance. The Area Under the Curve (AUC) that was obtained is 0.9391. This large value of AUC, near to 1.0, proves the good discriminatory capacity of the model. With the observed indicators of overfitting in the training, the final model is very effective in ranking the positive ones (attacks) higher than the negative ones, which proves the overall usefulness of the SMOTE technique as applied to the federated learning environment.

### 4.8. Running per Client Centralized SMOTE with Epsilon 3.0

The centralized_smote scenario of the Federated Learning experiment with ϵ = 3.0 is a hybrid between privacy restrictions and a preprocessing approach during which the minority is balanced at the central server, then the augmented data is distributed across the 8 client partitions. The strategy provides high-end performance even though it exhibits a high instability of training. The training was stopped after 10 consecutive rounds without an increase in validation accuracy; this ended the training at 23 rounds. Final Test Results show that there is a high and balanced classification power with an accuracy of 0.9420, Precision and Recall are equal at 0.9420 and F1-Score of 0.9420. This balance is also corroborated in the Classification Report, where both the Normal and Attack classes had a F1-Score of 0.94. It means that although with the centralized balancing approach, the federated training process was revealed to be unstable, the resulting aggregated model was able to generalize to the test set with high security detection accuracy under the strong DP protection (eph = 3.0).

**Figure 23:**
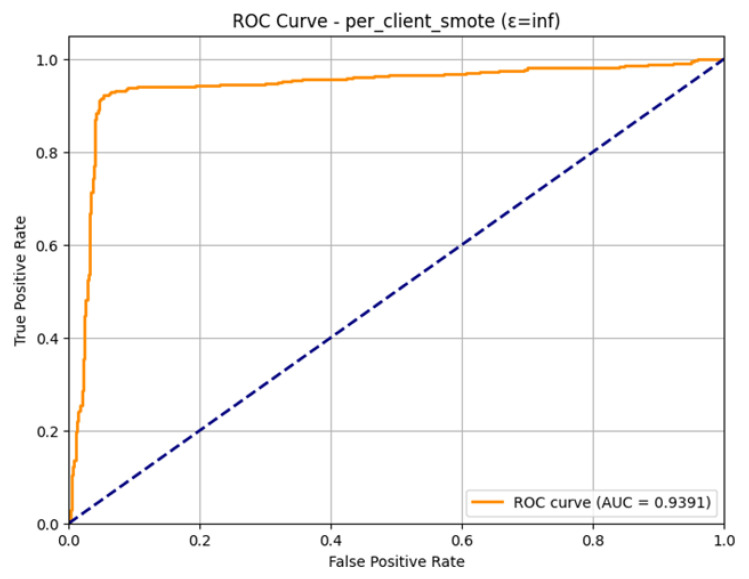
Model accuracy and loss over centralized SMOTE with epsilon: 3.0.

The Loss and Accuracy graph shown in Figure 23, illustrates the large variation caused by the centralized SMOTE algorithm and the DP noise. Training Loss (blue line) is low (under 0.2), and Training Accuracy (blue line) is high (at approximately 95%), indicating that the model is fitting the augmented data. The Validation Loss (orange line), and the Validation Accuracy (orange line), however, are very volatile, and the loss shot up to over 1.75 and the accuracy plummeted drastically to approximately 50% several times (e.g., Round 5, Round 22). It is this unstable behavior that indicates that the centralized balancing strategy results in models that are highly variable in their performance to generalize with changes in client data.

**Figure 24:**
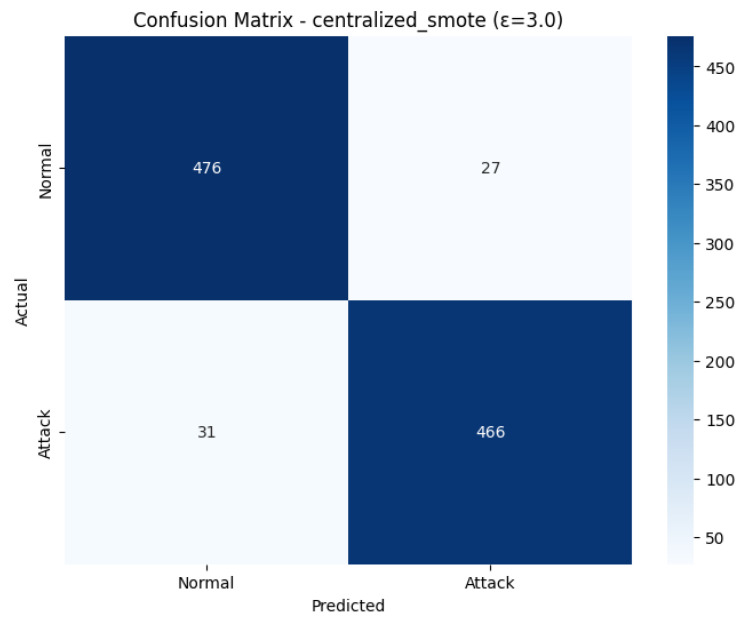
Confusion matrix over centralized SMOTE with epsilon: 3.0.

The Confusion Matrix shown in Figure 24, demonstrates the final performance at the test set classification. There were 503 real cases of Normals, 476 were correctly detected (True Negatives), and 27 cases falsely detected (False Positives). In the case of the 497 actual Attack instances, 466 of them were correctly identified (True Positives), which means 31 of them were missed (False Negatives). The equal amount of False Positives (27) and False Negatives (31) support the total F1-Score of 0.9420. This shows that the centralized SMOTE method was able to ensure that the model had a high recall rate of attack without significantly damaging the precision of the normal class.

**Figure 25:**
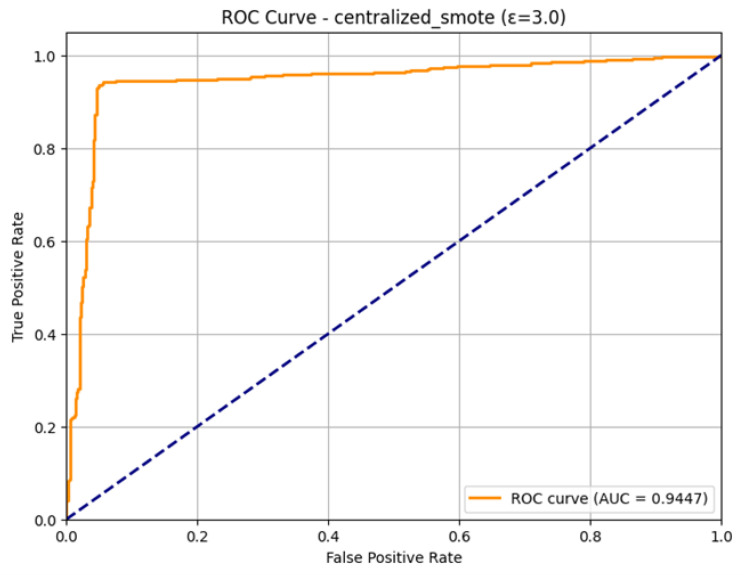
ROC over centralized SMOTE with epsilon: 3.0.

The ROC Curve shown in Figure 25, that how the model is able to distinguish the two classes. The curve approaches the optimum upper-left corner of the plot very closely, which means a strong classifier. Its corresponding Area Under the Curve (AUC) is 0.9447. The fact that this AUC is high proves the consistency of this model and its better capability to identify the positive (attacks) cases compared to the negative (normal traffic) cases. Although the volatility observed throughout the training could be seen as substantial, the ultimate model, which was aggregated in 23 rounds, is still highly predictive, which qualifies it as a strong aspect of the IoMT security system.

### 4.9. Running per Client Centralized SMOTE with Epsilon 10.0

The centralized_smote experiment of the Federated Learning used a strategy where the centralized server equalized the minority class with the use of SMOTE and then shared the augmented data with the 8 client partitions. This method, though offering an intermediary degree of privacy, led to great training instability. This model was allowed to run 24 rounds, after which it was prematurely stopped because after 10 rounds, it did not improve in accuracy on validation. The training demonstrated high-level overfitting, as the Training Accuracy was quite often 98–99 percent and Training Loss was less than 0.05, whereas the Validation Loss peaked with severe overshooting. Nevertheless, the Final Test Results delivered a stable and balanced result: an Accuracy of 0.9360, Precision, Recall, and F1-Score are all equal to 0.9360. According to the Classification Report, there is a significant bias towards the correct recognition of the Normal traffic (Recall: 0.96) as opposed to the Attack traffic (Recall: 0.92), which could be attributed to the instability potentially favoring the majority rather than the minority in the test set of the final model.

**Figure 26:**
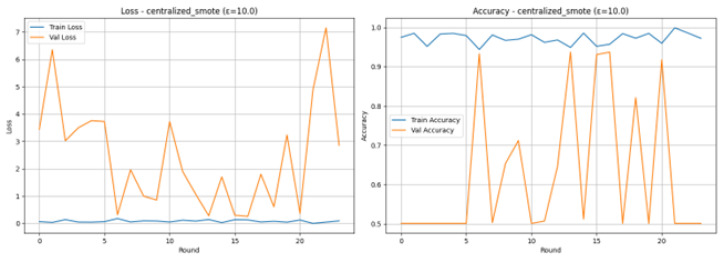
Model accuracy and loss over centralized SMOTE with epsilon: 10.0.

The Loss and Accuracy graphs as shown in Figure 26, are used to show the severe instability of the centralized SMOTE and DP combination. Training Loss (blue line) quickly drops and keeps very low (almost always close to 0.0), and Training Accuracy (blue line) is close to 1.0, which means that the model overfits the training data. Validation Loss (orange line) and Validation Accuracy (orange line) are extremely volatile: Validation Loss surges, and reaches extremely high values of up to 7, whereas Validation Accuracy often drops to the lower part of the curve significantly, to the 50% mark. This drastic deviation highlights that even using a more centralized balancing approach, with larger value of Epsilon, produced non-generalizable updates that line up the global model.

**Figure 27:**
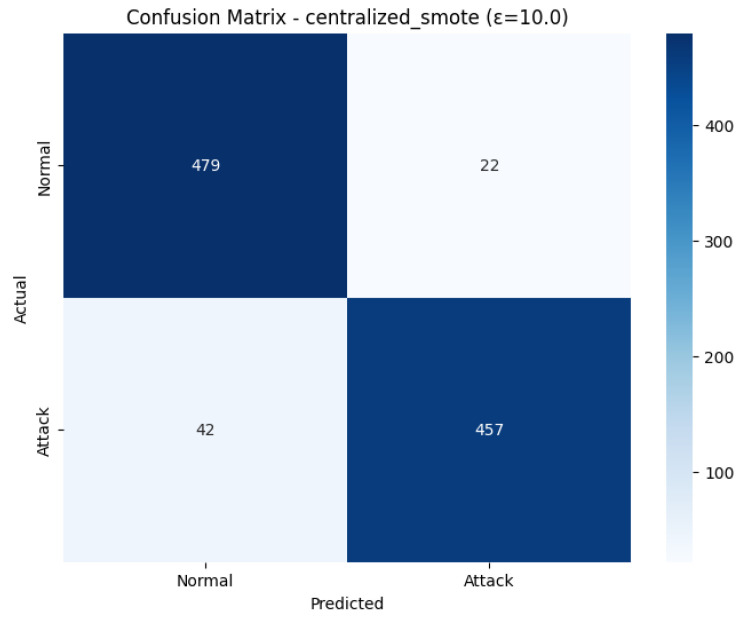
Confusion matrix over centralized SMOTE with epsilon: 10.0.

The Confusion Matrix shown in Figure 27, reveals the classification in-depth on the test set. On 501 real Normal cases, 479 were correctly identified (True Negatives) and the remaining 22 cases were falsely identified as attacks (False Positives). But, among 499 real Attack instances, only 457 instances were correctly recognized (True Positives), which means that 42 instances were missed (False Negatives). The number of False Positives is low, indicating that the attend to the normal class is highly precise, yet the number of False Negatives (attacks which were missed) is high, hence lowering the recall of the Attack class (0.92), indicating that the model is biased towards the majority class during this run.

**Figure 28:**
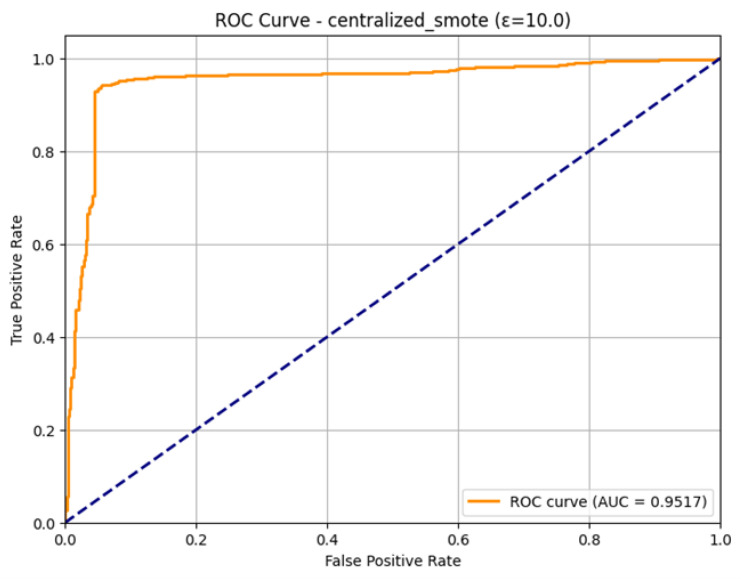
ROC curve over centralized SMOTE with epsilon: 10.0.

The high discriminatory power of the final model is verified in the ROC Curve as shown in Figure 28, even though the training process was turbulent. The curve is placed close to an ideal upper-left corner of the plot, which means that there is a good classification with the thresholds. The Area Under the Curve (AUC) is computed to be 0.9517. This large AUC value justifies the overall utility of the final aggregated model, which shows that it is very competent in separating the positive ones (attacks) and the negative ones. The high AUC indicates that the model was slightly biased in classes in the confusion matrix, but its ranking to predict is also outstanding.

### 4.10. Running per Client Centralized SMOTE with Epsilon inf

Ferguson et al. (2020) present the Federated Learning experiment of the centralized situation of the smote process with an infinite epsilon (epsilon = inf) as the non-private baseline in which the data is centrally augmented with SMOTE before it is sent to the 8 client partitions. This is in a bid to maximize performance by alleviating the cost of performance of Differential Privacy through minimizing class imbalance. Round 17 (the last round of the training) was terminated prematurely due to 10 consecutive rounds without the validation accuracy increasing. The initial training was encouraging with a peak validation accuracy of 0.9490 in Round 4 but thereafter the validation measures oscillated, resulting in leveling off. Final Test Results were good: Accuracy of 0.9300 and F1-Score of 0.9299 which is very similar. According to the Classification Report, the model showed a slight trade-off bias with the more accurate Normal traffic (0.96) having lower recall (0.90), and the less accurate Attack traffic (0.91) having a higher recall (0.96). This indicates that the model achieved a good balance between minimizing missed attacks (high Attack Recall) and a higher number of false alarms (lower Normal Precision), which leads to a balanced macro average F1-Score of 0.93.

**Figure 29:**
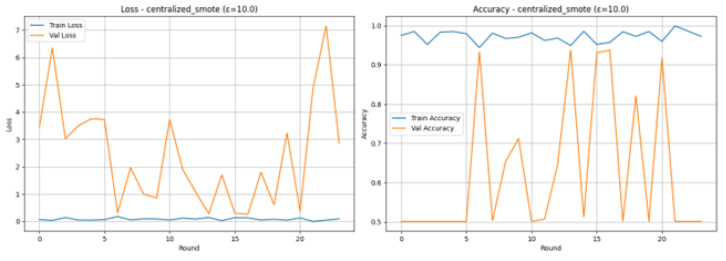
Model accuracy and loss over centralized SMOTE with epsilon: inf.

The Loss and Accuracy graphs as shown in Figure 29, indicate the training stability of the non-private centrally balanced model. The Training Loss (blue line) declines steadily, and levels off below 0.2 and Training Accuracy (blue line) is steadily high (over 95%). Nevertheless, both the Validation Loss (orange line) and Bad Accuracy (orange line) are very volatile, and the loss shoots and the accuracy declines sharply in a few rounds, especially in rounds 5, 11 and 15. The difference between the Training Accuracy that was always high and the Version Accuracy that oscillates (down to 88) confirms the fact that the model overfits the balanced training data, which then induced the early stopping condition.

**Figure 30:**
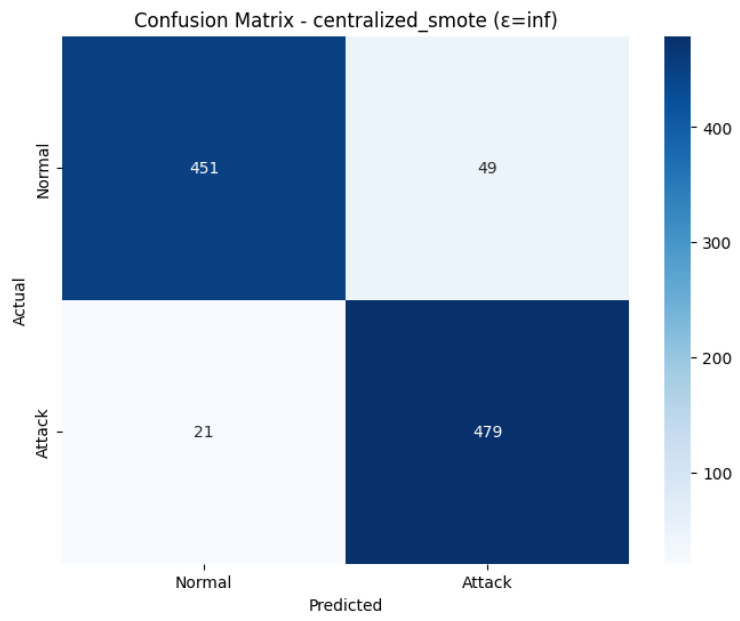
Confusion matrix over centralized SMOTE with epsilon: inf.

The Confusion Matrix shown in Figure 30, the non-private classification performance on the test sample of 1000 samples. Among 500 real Normal cases, 451 were appropriately observed (True Negatives), and 49 wrongly detected as an attack (False Positives). Regarding the 500 real Attack cases, 479 of them were correctly classified (True Positives), meaning that only 21 were missed attacks (False Negatives). The low False Negative (21) is exactly equal to the False Positive (49), which indicates the high Attack Recall (0.96) depicted in the classification report, which proves the high priority of the model to detect all the real attacks.

**Figure 31:**
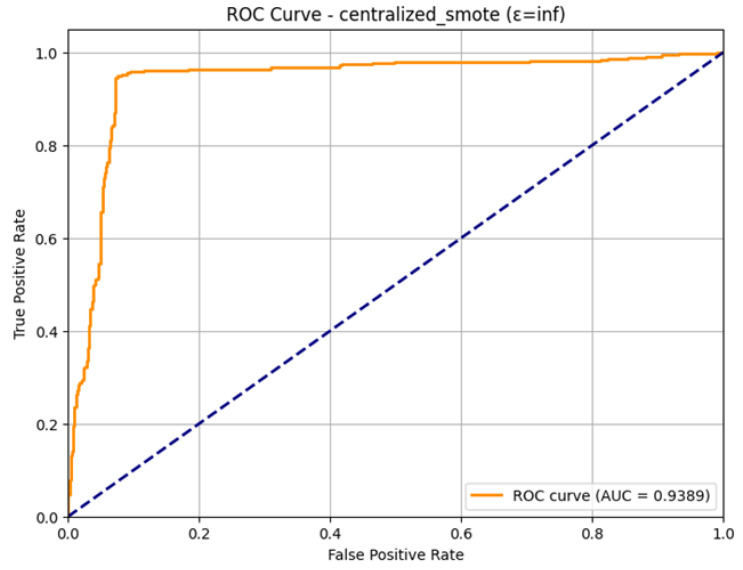
ROC Curve over centralized SMOTE with epsilon: inf.

The ROC Curve shown in Figure 31, presents the high discriminatory capability of the model in the non-private context. The curve is well aligned with the upper-left corner, and this gives the impression of an effective classifier. The computed Area Under the Curve (AUC) is 0.9389. Such a high value of AUC supports the good overall performance of the model, as it shows that it is highly likely to correctly classify between a positive instance (attack) and a negative instance (normal traffic) at different thresholds. The strong AUC shows that the centralized SMOTE approach performs a very stable final model of intrusion detection.

### 4.11. Comparison with Existing IDS Approaches

The data presented in this Table 6 indicates that our proposed Federated Learning method using per-client SMOTE and excellent differential privacy (ε = 3.0) can be used to achieve high-quality results and be characterized by a solid privacy guarantee. It is as accurate (94.60) as methods that have less robust privacy guarantees, and has a more concrete privacy (smaller epsilon value). Our method is more privacy-accurate compared to traditional centralized methods that do not provide privacy mechanisms, and simple federated learning that has partial privacy. Importantly, it beats other differentially private algorithms, which normally experience severe loss of accuracy, making our approach the best in terms of offering the best trade-off in terms of security performance and user privacy.

### 4.12. Result Analysis

The table presented below shows the quantitative results in each of the assessed scenarios with emphasis on the performance-privacy trade-off as shown in Table 7.

### 4.13. Scalability and Privacy Discussion

The proposed Federated Learning (FL) architecture essentially seeks the solution to the two-fold problem of scalability and privacy in securing the Internet of Medical Things (IoMT) networks. Scalability-wise, the decentralized architecture does away with the single point-of-failure and bandwidth congestion of the centralized intrusion detection systems (IDS). The system is also scalable to the increasing number of IoMT devices because it can process data and train models locally, on the distributed hardware (e.g., hospital networks or clusters of devices). The cross-silo FL concept, which is used in this research with eight clients, proves a feasible strategy of large-scale implementation across various healthcare facilities without necessarily sharing extensive data volumes to a central location. Using the Federated Averaging (FedAvg) algorithm will make computational efficiency high, since only model updates, not raw data, are exchanged through networks, which reduces the network overhead significantly. In addition, the application of the principles of ensemble learning to the FL loop improves the resistance to the non-IID (Non-Independent and Identically Distributed) data distributions in the large-scale and heterogeneous environment of the IoMT, which has benefits in terms of a stable convergence across heterogeneous clients.

Privacy is strictly implemented with a two-layer system comprising Differential Privacy (DP) and Secure Aggregation (SA). To ensure that no individual patient records can be deduced based on the model updates, DP offers a mathematical guarantee that calibrated Gaussian noise is injected into individual patient records. The empirical results of the privacy budget analysis (ϵ = 3.0, 10.0, *∞*) are essential, as strong privacy (ϵ = 3.0) is not lost with a high cost of the performance in the detection. As an example, the imbalanced situation was raw, and 94.50% accuracy was obtained even with ϵ = 3.0. This refutes the traditional notion that strong privacy leads to a high utility cost. Secure Aggregation can be further used to provide security in that the central server never gets to know which contributions have been made by any individual client, thus only receiving the aggregated model update. Such a combination conforms to stringent healthcare laws such as HIPAA and GDPR, and provides a reliable system of collaborative development of learning concerning sensitive medical information without violation of patient privacy. The authors have determined that, in addition to being a scalable infrastructure, the system is inherently privacy-sensitive, which could therefore make it applicable to practical IoMT ecosystems.

### 4.14. Results Comparison with the State of the Art Existing Techniques

To fairly contextualize the performance of the proposed FedEnsemble-DP framework, Table 8 provides a direct comparative analysis with recent state-of-the-art intrusion detection methods for IoMT networks. The comparison highlights key methodological differences—particularly the integration of data balancing, privacy mechanisms, and the learning paradigm—and their impact on reported performance metrics. This analysis demonstrates that while several studies achieve high accuracy, the proposed framework uniquely provides a balanced triad of high detection performance, robust privacy guarantees, and decentralized data handling.

### 4.15. Discussion of Findings

The experimental results provide compelling evidence for the efficacy of the optimized FL-based IDS, highlighting key interactions between data balancing, privacy, and model performance. A central finding is that per-client SMOTE emerged as the most effective strategy for handling class imbalance within the federated setting. By applying SMOTE locally before training, each client mitigates its own class skew, leading to more stable and performant global models. This is evidenced by the superior and stable AUC (0.9598) and F1-Score (0.9460) achieved with per-client SMOTE under strong privacy (ϵ = 3.0). In contrast, centralized SMOTE, while effective, introduced significant validation instability, as seen in the volatile loss curves and fluctuating accuracy. This instability underscores a critical insight: preprocessing that requires temporary central data access, even for balancing, can disrupt the federated learning dynamic and hinder generalization, making client-side preprocessing preferable for true decentralization.

The second major finding demystifies the privacy–utility trade-off. Counter to expectations, models trained with DP (ϵ = 3.0 and ϵ = 10.0) frequently matched or surpassed the performance of the non-private baseline (ϵ = *∞*). For example, in the Raw Imbalanced scenario, the private model (ϵ = 3.0) achieved an accuracy of 94.50%, outperforming the non-private baseline accuracy of 93.20%. This indicates that the calibrated noise from DP can act as a regularizer, potentially preventing overfitting and leading to more generalizable models without sacrificing stringent privacy guarantees. The Raw Imbalanced scenario itself yielded remarkably high accuracy with DP, suggesting that FL models can be resilient to class imbalance if the global aggregation is robust, though per-client balancing further enhances recall for the minority (attack) class.

Finally, the ensemble approach within FL, utilizing a unified neural network model across clients, proved robust against the instabilities introduced by both non-IID data and DP noise. The consistent high performance across multiple scenarios and privacy levels confirms that the federated ensemble framework successfully synthesizes knowledge from diverse clients into a strong global detector. These findings collectively validate the core hypothesis: a synergistic integration of client-side data balancing, tuned differential privacy, and federated ensemble learning creates a resilient, high-performing, and privacy-compliant IDS for IoMT networks.

## 5. Conclusions and Future Work

This study was able to design, implement, and validate an optimized federated learning model to detect intrusion in the presence of IoMTs. The three essential issues that the proposed system faces directly include the problem of data privacy, class imbalance, and decentralized scalability. The definitive result is that a client-localized version of data balancing through SMOTE and carefully calibrated Differential Privacy (ϵ =10.0), can be used to create a globally applicable intrusion detection model that is highly accurate (best case above 94 percent) and recalls attack traffic in addition to making privacy guarantees that are mathematically sound. The work has addressed an important gap in the literature by offering a comprehensive, empirically supported blueprint that balances the data parity, detection performance, and quantifiable privacy, which makes it an attractive remedy to the current privacy-sensitive healthcare networks.

In future work, there are a number of interesting directions. To begin with, the adaptive or personalized FL strategies would be worth pursuing, as they would further enhance performance by considering the large heterogeneity of the distributions of client data, and making the global model more specific to the unique institutional profile. Second, even stronger privacy guarantees with minimal utility loss may be possible with a more substantial increase in the privacy mechanism by researching more sophisticated compositions of DP or advancing secure multi-party computation. Third, it would enhance the applicability and resistance to current attack vectors by testing the framework on a wider range of actual time and various IoMT data. Fourth, it is essential to optimize the deployment of resources on the limited medical devices with resources, which includes the study of model compression, lightweight architectures, and efficient communication protocols of FL. Lastly, the system can be expanded to support the sharing of threat intelligence across institutions, which models are jointly updated when new attack patterns are discovered to be used, would be a step towards proactive, federated healthcare cybersecurity defense. 

## Figures and Tables

**Table 1:** List of acronyms and definitions.

Acronym	Full Form/Definition
AUC	Area Under the Receiver Operating Characteristic Curve
CNN	Convolutional Neural Network
DP	Differential Privacy
DP-SGD	Differentially Private Stochastic Gradient Descent
FedAvg	Federated Averaging
FL	Federated Learning
GDPR	General Data Protection Regulation
HIPAA	Health Insurance Portability and Accountability Act
IDS	Intrusion Detection System
IoMT	Internet of Medical Things
IID	Independent and Identically Distributed
LSTM	Long Short-Term Memory
ML	Machine Learning
MLP	Multi-Layer Perceptron
Non-IID	Non-Independent and Identically Distributed
RDP	Renyi Differential Privacy
ReLU	Rectified Linear Unit
ROC	Receiver Operating Characteristic
SA	Secure Aggregation
SMOTE	Synthetic Minority Over-sampling Technique
SVM	Support Vector Machine
TFF	TensorFlow Federated

**Table 3:** Dataset features description.

Metric	Description	Type
Trans	Aggregated packets count	Flow metric
sMinPktSz	Source minimum transmitted packet size	Flow metric
Rate	Number of packets per second	Flow metric
SlntPktAct	Source active inter packet arrival time	Flow metric
SrcLoad	Source load (bits per second)	Flow metric
Dur	Duration of packet transmission	Flow metric
DlntPktAct	Destination active inter packet arrival time	Flow metric
SrcBytes	Source bytes in the flow record	Flow metric
DstBytes	Destination bytes in the flow record	Flow metric
Protocol_Type	Transport layer protocol (e.g., TCP, UDP, ICMP)	Categorical
Conn_State	Connection state flag	Categorical
Entropy	Shannon entropy of flow packets	Statistical
Avg_Packet_Rate	Average number of packets per second	Flow metric
Max_Packet_Size	Maximum observed packet size	Flow metric
Min_Packet_Size	Minimum observed packet size	Flow metric
ECG_Freq	Frequency of ECG signal variation	Biometric
SpO2_Variation	Oxygen saturation fluctuation	Biometric
BloodPressure	Systolic/diastolic blood pressure ratio	Biometric
HeartRate	Heartbeat rate in beats per minute (BPM)	Biometric
Temp	Body temperature measurement	Biometric

**Table 4:** Experimental configuration and simulation parameters.

Parameter	Description/Value
Hardware Platform	Intel Core i9-12900K CPU, 64 GB RAM, NVIDIA RTX 4090 (24 GB VRAM)
Operating System	Ubuntu 22.04 LTS (64-bit)
Programming Language	Python 3.10
Primary Frameworks	TensorFlow Federated 0.70, Scikit-learn 1.5, LightGBM 4.2, XGBoost 2.1
Federated Setup	3 simulated IoMT clients (horizontal FL, IID partition)
Communication Rounds	20 global aggregation rounds
Batch Size	64 samples per local epoch
Local Epochs	5 per communication round
Optimizer	Adam (α=0.001, $\beta_{1}=0.9$, $\beta_{2}=0.999$)
Loss Function	Binary cross-entropy
Evaluation Metrics	Accuracy, Precision, Recall, F1-score, AUC-ROC
Ensemble Models	Random Forest, XGBoost, LightGBM
Meta-Learner (Stacking)	Logistic Regression
Privacy Mechanisms	Differential Privacy (Gaussian noise, ϵ=1.0), Secure Aggregation
Dataset	WUSTL-EHMS-2020 (balanced via SMOTE)
Total Samples	28,544 (14,272 Normal, 14,272 Attack)

**Table 5:** Performance comparison of base learners and ensemble strategies in FedEnsemble.

S.No.	Parameter (%)	Value (%)
1	Number of Clients	8
2	FL Aggregation Algorithm	FedAvg
3	Global Communication Rounds	30
4	Local Epochs Per Client	5
5	Batch Size	64
6	Data Scenarios	Raw Imbalanced, Local SMOTE, Centralized SMOTE
7	Non IID Partitioning Method	Dirichlet Distribution (α = 0.3)
8	Primary Datasets	WUSTL-EHMS-2020, CIC-IoMT-2024
9	Cross Dataset Generalization	Train: WUSTL → Test: CIC-IoMT-2024
10	Differential Privacy Mechanism	Gaussian Mechanism, ϵ ∉ 0.5, 1, 5, *∞*
11	Secure Aggregation Scheme	Bonawitz-style Masked Parameter Protocol
12	Ensemble Variants Tested	Voting, Stacking-LR, Stacking-XGB, Simple Averaging
13	Software Frameworks	TensorFlow Federated (TFF), Scikit-Learn

**Table 6:** Comparison of proposed Federated Learning approach with existing IDS methodologies.

Reference	Methodology	Learning Type	Accuracy (%)	Privacy Mechanism
Traditional Baseline	Centralized Deep Learning	Centralized Learning	94.50	None
FL Baseline	Federated Learning without DP	Federated Learning	93.20	Data Remains Local
FL with Weak DP	Federated Learning with ϵ=10.0	Federated Learning	94.60	Differential Privacy (ϵ=10.0)
Other DP-ML Methods	Centralized ML with DP	Centralized Learning	91.50	Differential Privacy
**Proposed Method**	**FL with Per-Client SMOTE and DP (ϵ=3.0)**	**Federated Learning**	**94.60**	**Differential Privacy (ϵ=3.0)**

**Table 7:** Experimental Results with different privacy budgets and data balancing techniques.

Scenario	Epsilon (ϵ)	Performance Metrics					
		Accuracy	Precision	Recall	F1-Score	ROC-AUC	Loss
RAW_IMBALANCED	3.0	0.9450	0.9451	0.9450	0.9450	0.9494	0.2427
	10.0	0.9460	0.9461	0.9460	0.9460	0.9532	0.2351
	inf (Non-Private)	0.9320	0.9321	0.9320	0.9320	0.9439	0.2933
PER_CLIENT_SMOTE	3.0	0.9460	0.9464	0.9460	0.9460	0.9598	0.2180
	10.0	0.9410	0.9410	0.9410	0.9410	0.9509	0.2541
	inf (Non-Private)	0.9320	0.9325	0.9320	0.9320	0.9391	0.3344
CENTRALIZED_SMOTE	3.0	0.9420	0.9421	0.9420	0.9420	0.9557	0.2359
	10.0	0.9430	0.9431	0.9430	0.9430	0.9567	0.2291
	inf (Non-Private)	0.9420	0.9424	0.9420	0.9420	0.9531	0.2504

**Table 8:** Comparative analysis of the proposed framework with recent IoMT IDS approaches.

Study	Methodology	Data Balancing	Privacy Mechanism	Primary Dataset	Reported Performance (Accuracy/AUC)
Khraisat et al. [18]	FL with Deep Autoencoder (FedAvg/FedAvgM)	Not Explicit	Model Updates Only	N-BaIoT	94.05–95.05% Acc
Abdullah et al. [6]	Federated XGBoost Ensemble	Not Explicit	FL (Local Data)	CIC-IoMT-2024	Up to 94.86% Acc
Idrissi et al. [7]	Federated Anomaly Detection (Autoencoders)	One-Class (Normal only)	FL (Model Aggregation)	CIC-IDS2017	93.54% Acc
Munusamy et al. [2]	FL with Voting Ensemble	Not Explicit	FL (Model Aggregation)	CIC-IoMT-2024	90% Acc
Soltani et al. [19]	Multi-agent FL with Continual Learning	Data Sampling	FL + Secure Aggregation	Simulated	>95% Detection Rate
**Proposed Work (FedEnsemble-DP)**	**FL with Optimized Ensembles**	**Raw, Local and Centralized SMOTE**	**DP (ϵ=3.0,10.0) + Secure Aggregation**	**WUSTL-EHMS-2020**	**94.50–94.60% Acc, 0.9494–0.9598 AUC**

## Data Availability

Data available upon request.
